# LLO-mediated Cell Resealing System for Analyzing Intracellular Activity of Membrane-impermeable Biopharmaceuticals of Mid-sized Molecular Weight

**DOI:** 10.1038/s41598-018-20482-2

**Published:** 2018-01-31

**Authors:** Masataka Murakami, Fumi Kano, Masayuki Murata

**Affiliations:** 1 Department of Life Sciences, Graduate School of Arts and Sciences, The University of Tokyo, 3-8-1 Komaba, Meguro-ku, Tokyo 153-8902 Japan; 2 Cell Biology Center, Institute of Innovative Research, Tokyo Institute of Technology, 4259 Nagatsuta, Midori-ku, Yokohama, Kanagawa 226-8503 Japan; 3 Laboratoty of Frontier Image Analysis, Graduate School of Arts and Science, The University of Tokyo, 3-8-1 Komaba, Meguro-ku, Tokyo 153-8902 Japan

## Abstract

Cell-based assays have become increasingly important in the preclinical studies for biopharmaceutical products such as specialty peptides, which are of interest owing to their high substrate specificity. However, many of the latter are membrane impermeable and must be physically introduced into cells to evaluate their intracellular activities. We previously developed a “cell-resealing technique” that exploited the temperature-dependent pore-forming activity of the streptococcal toxin, streptolysin O (SLO), that enabled us to introduce various molecules into cells for evaluation of their intracellular activities. In this study, we report a new cell resealing method, the listeriolysin O (LLO)-mediated resealing method, to deliver mid-sized, membrane-impermeable biopharmaceuticals into cells. We found that LLO-type resealing required no exogenous cytosol to repair the injured cell membrane and allowed the specific entry of mid-sized molecules into cells. We use this method to introduce either a membrane-impermeable, small compound (8-OH-cAMP) or specialty peptide (Akt-in), and demonstrated PKA activation or Akt inhibition, respectively. Collectively, the LLO-type resealing method is a user-friendly and reproducible intracellular delivery system for mid-sized membrane-impermeable molecules into cells and for evaluating their intracellular activities.

## Introduction

Cell-based assays have become increasingly important in preclinical studies for drug discovery. Such assays enable the detailed study of the mechanisms of drug action, speeding up development time and reducing costs. Recently, biopharmaceutical products such as nucleotides, peptides, and antibodies have received increased attention owing to their higher substrate specificities and are thought to overcome certain disadvantages of small-molecule compounds^1–3^. In particular, mid-sized peptides (less than ~10 kDa) can be chemically synthesized, unlike antibodies, and are expected to reduce the cost in development and production of drugs. One example is CP2, a cyclic peptide inhibitor of histone demethyrase^4^, which is a modified, cyclic compound comprising natural and unnatural amino acids. However, for intracellular targets, very high concentrations of proteins and cytoskeletal or membranous structures in the cells might affect the activity that was measured in the *in vitro* system^5–7^, which is a critical issue for drug efficacy and design. Additionally, such mid-size products are generally membrane impermeable and methods to introduce them into cells have also been extensively studied^8,9^. Thus, to test their efficacy, these products should be introduced into cells across the plasma membranes and their activity should be evaluated *in situ* in cells.

Several methods for introducing molecules into cells have been developed: microinjection^10,11^, electroporation^12^, cell-penetrating peptides (CPPs)^13^. There are both advantages and disadvantages to each method. Microinjection can be performed using commercially available equipment, but may be difficult to apply to high-content analyses. Recent advances in electroporation enable delivery of various types of molecules such as proteins, nucleotides, and small chemical compounds into cells using dedicated equipment, but it is inadequate for large-scale studies and can cause damage to cells. CPPs are peptides of typically 5–30 amino acids that can facilitate uptake of linked cargo into cells. CPP-based delivery of molecules into cells is less toxic, allowing its therapeutic use, but CPP conjugation to cargo molecules is required, which might perturb the cargo’s function.

We previously described a “cell-resealing technique” that makes use of the temperature-dependent pore-forming activity of the streptococcal toxin, streptolysin O (SLO), to introduce various molecules into cells^14^. SLO is a cholesterol-dependent cytolysin (CDC) derived from *Streptococcus pyogenes*, and enables the plasma membrane to be permeabilized selectively with minimal damage to the membranes of intracellular organelles. The size of the pore formed by SLO is about ~30 nm in diameter^15^, and various water-soluble molecules such as antibodies and 2,000 kDa dextran can be introduced into cells^14^. This method can also be used to remove endogenous cytosol so as to enable the manipulation of the intracellular environment. Interestingly, the injured plasma membrane of the permeabilized cells can be rapidly repaired and resealed within a few minutes by incubation of cells with cytosol and calcium ions^16–18^. Hereafter, we refer to this resealing technique as the SLO-type resealing method.

As SLO-type resealing can manipulate 1 ~ 10^6^ adherent or suspension cells at once, the resealed cells can be analyzed by various microscopic and biochemical methods. No specific modification of the biomolecules to be introduced by induced membrane permeability is required. The pores formed by SLO remove endogenous cytosol and enable the manipulation of the intracellular environment. For example, by replacing HeLa cell cytosol with that from diabetic mouse liver, we established a diabetic model cell, and identified several perturbations of endocytic pathways that occur specifically under diabetic conditions^14^. However, when the SLO-type resealing method is applied to delivery of molecules into cells, leakage of the endogenous cytosol is undesirable. Furthermore, some types of cells such as non-immortalized cells are not suitable for cytosol preparation because of the difficulty to culture them at large scale, we cannot always obtain cytosol derived from the same type of cells with resealed cells used for experiments for repairing the injured plasma membrane.

To optimize the cell-resealing method for drug screening, we focused on the toxin listeriolysin O (LLO) produced by *Listeria monocytogenes*. LLO is a temperature-sensitive CDC like SLO^19^, but has an unusual feature in that it prefers an acidic environment for optimal activity^20^. At neutral pH, LLO on membrane structures produces only small lesion, through which only small molecules such as ions can pass^21,22^. Therefore, we proposed to use LLO at neutral pH as an alternative toxin in the resealing method for SLO, so as to introduce only small and mid-sized molecules into the cell with minimum leakage of endogenous cytosol. In addition, under neutral or alkaline conditions at >30 °C, LLO in solution self-aggregates due to a change in conformation, and it thereby loses cytotoxic activity^20^. This suggests that the perforation activity of LLO could be regulated by temperature, which would be convenient so as to inactivate LLO after cell-resealing and potentially alleviate any additional damage that could affect the subsequent biochemical analysis.

In this study, we developed a new resealing method named LLO-type resealing. In this method, LLO can permeabilize the plasma membranes, and deliver soluble low- and mid-sized molecules into cells. Compared to SLO-type resealing, LLO-type one did not require cytosol to repair the plasma membrane. Furthermore, we show the utility of the LLO-type resealing method for biochemical analyses by demonstrating that a membrane-impermeable PKA activator, 8-OH-cAMP, and an Akt-inhibitory peptide, Akt-in, are functional in the resealed cells. Collectively, the LLO-type resealing method is a user-friendly and reproducible system for the delivery of membrane-impermeable, mid-sized molecules into cells and is suitable for analyzing the intracellular functions of delivered molecules.

## Results and Discussion

### LLO permeablizes the plasma membrane of HeLa cells at neutral pH

**Figure 1:**
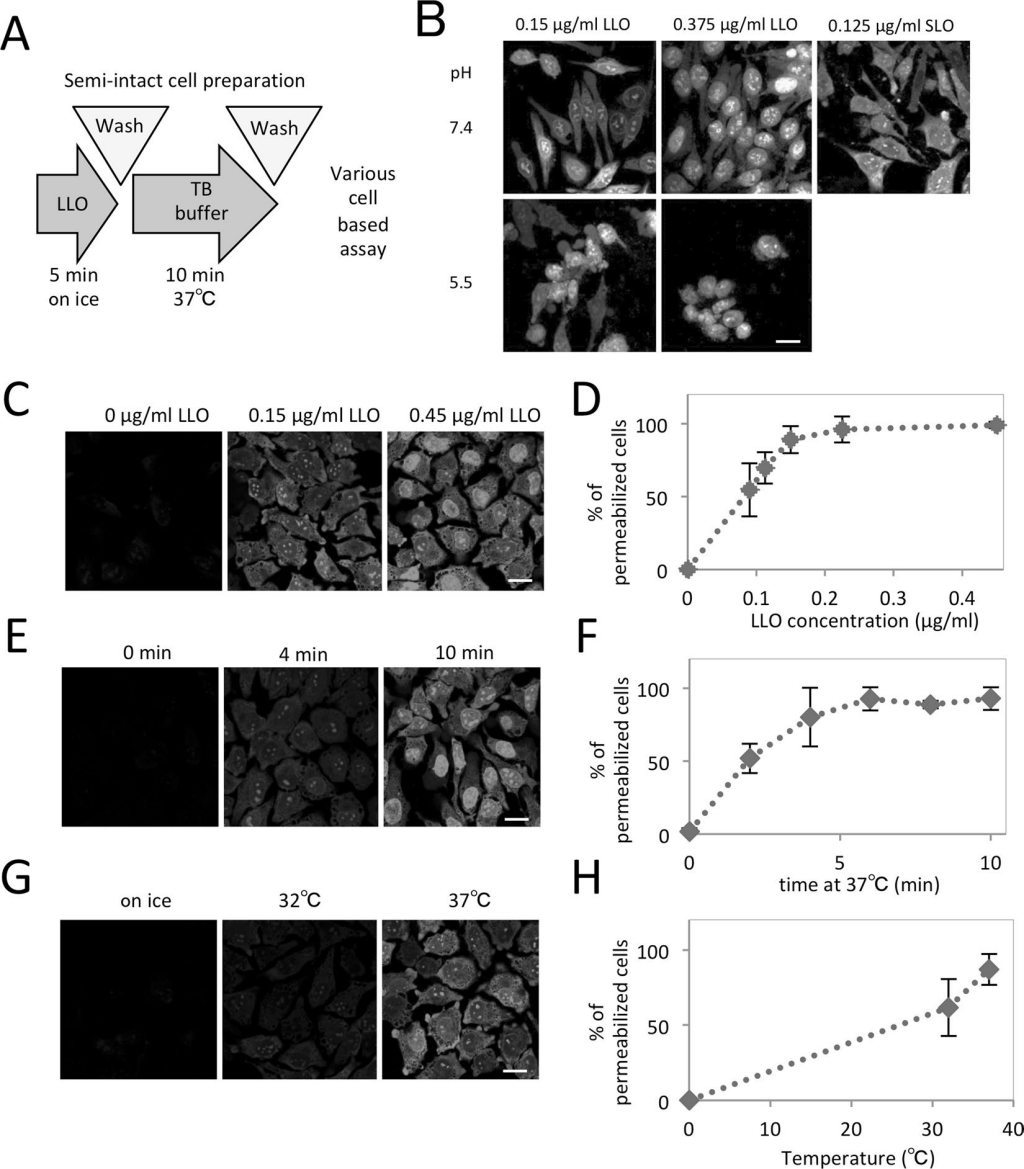
Permeabilization of HeLa cells with listeriaolysin O (LLO). (**A**) Basic protocol for permeabilization of HeLa cells with LLO. HeLa cells were incubated with 0.15 μg/ml LLO on ice for 5 min, and further incubated with prewarmed TB that contained propidium iodide at 37 °C for 10 min. (**B**) HeLa cells were incubated with 0.15 or 0.375 μg/ml LLO or 0.125 μg/ml SLO on ice for 5 min. After washing with PBS, the cells were further incubated with prewarmed TB that contained propidium iodide (PI) at pH 7.4 or pH 5.5 at 37 °C for 10 min, and were observed by confocal microscopy. Bar = 20 μm. (**C**,**D**) Dependency of permeabilization on LLO concentration. HeLa cells were treated with LLO at the concentrations of 0, 0.09, 0.15, 0.23 or 0.45 μg/ml on ice for 5 min, and were permeabilized with prewarmed TB that contained PI. The cells were observed by confocal microscopy (**C**). Bar = 20 µm. The number of PI-positive permeabilized cells was counted and the means and standard deviations for the percentage of PI-positive permeabilized cells are shown in the graph (**D**). (**E**,**F**) Dependency of permeabilization on the incubation time with TB. HeLa cells were treated with 0.15 µg/ml LLO on ice for 5 min, and further with prewarmed TB that contained PI at 37 °C for 0, 2, 4, 6, 8 or 10 min. The cells were observed by confocal microscopy (**E**). Bar = 20 µm. The means and standard deviations for the percentage of PI-positive permeabilized cells are shown in the graph (**F**). (**G**,**H**) Dependency of permeabilization on the temperature of TB incubation. HeLa cells were treated with 0.15 µg/ml LLO on ice for 5 min, and further with prewarmed TB that contained PI at 0, 32 or 37 °C for 10 min. The cells were observed by confocal microscopy (**G**). Bar = 20 µm. The means and standard deviations for the percentage of PI-positive permeabilized cells are shown in the graph (**H**).

We first examined whether LLO could induce permeabilization of HeLa cells at neutral or acidic pH using propidium iodide (PI), which is a membrane-impermeable dye for nucleic acids (MW = 668). The basic protocol of LLO treatment is shown in Fig. 1A. Briefly, HeLa cells were treated with 0.15 or 0.375 µg/ml LLO or 0.125 µg/ml SLO on ice for 5 min. After washing with transport buffer (TB) on ice three times, the cells were incubated with pre-warmed TB of pH 5.5 or 7.4 containing PI at 37 °C for 10 min. Confocal microscopy revealed that, at pH 5.5, LLO permeated the cell membrane, which was confirmed by staining of cells (mainly within the nucleus) with PI, but caused severe damage to cells (Fig. 1B). Interestingly, PI staining was also observed in cells treated with LLO at neutral pH (Fig. 1B), which is reported to reduce the ability of LLO to permeabilize the membrane^19,20^. We observed more uniform staining by PI and less morphological damage in LLO-treated cells, compared to SLO-treated cells at pH 7.4.

Next, we examined the effect of LLO concentration, incubation time, and incubation temperature on permeabilization of the cell membrane at pH 7.4. Permeabilization efficiency was estimated by the percentage of cells stained with PI. We examined the permeabilization efficiency of cells treated with 0, 0.09, 0.13, 0.15, 0.23, 0.45 µg/ml of LLO, and found that greater than ~90% of cells were permeabilized at concentrations of 0.15 µg/ml LLO (26 nM, 1.9 × 10^2^ HU/ml) and above (Fig. 1C,D). The effect of incubation time was also examined, and the permeabilization efficiency increased in a time-dependent manner from 0 to 6 min at which point it became stable (Fig. 1E,F). As the pore-forming activity of LLO at pH 7.4 is reported to be inactive at 37 °C after greater than a 10 min incubation^20^, the incubation time for LLO-mediated permeabilization was fixed at 10 min at pH 7.4 at 37 °C to avoid the influence of residual LLO in downstream processes. As shown in Fig. 1G,H, the permeabilization efficiency increased with temperature, and it was achieved sufficiently at 37 °C after 10 min incubation. Taken together, we set the optimal condition for LLO-mediated permeabilization for HeLa cells as follows: cells were incubated with 0.15 µg/ml LLO on ice for 5 min, and further with TB at 37 °C for 10 min. This experimental condition was used for the subsequent experiments.

### LLO-type resealing does not require exogenous cytosol but does require ATP

**Figure 2:**
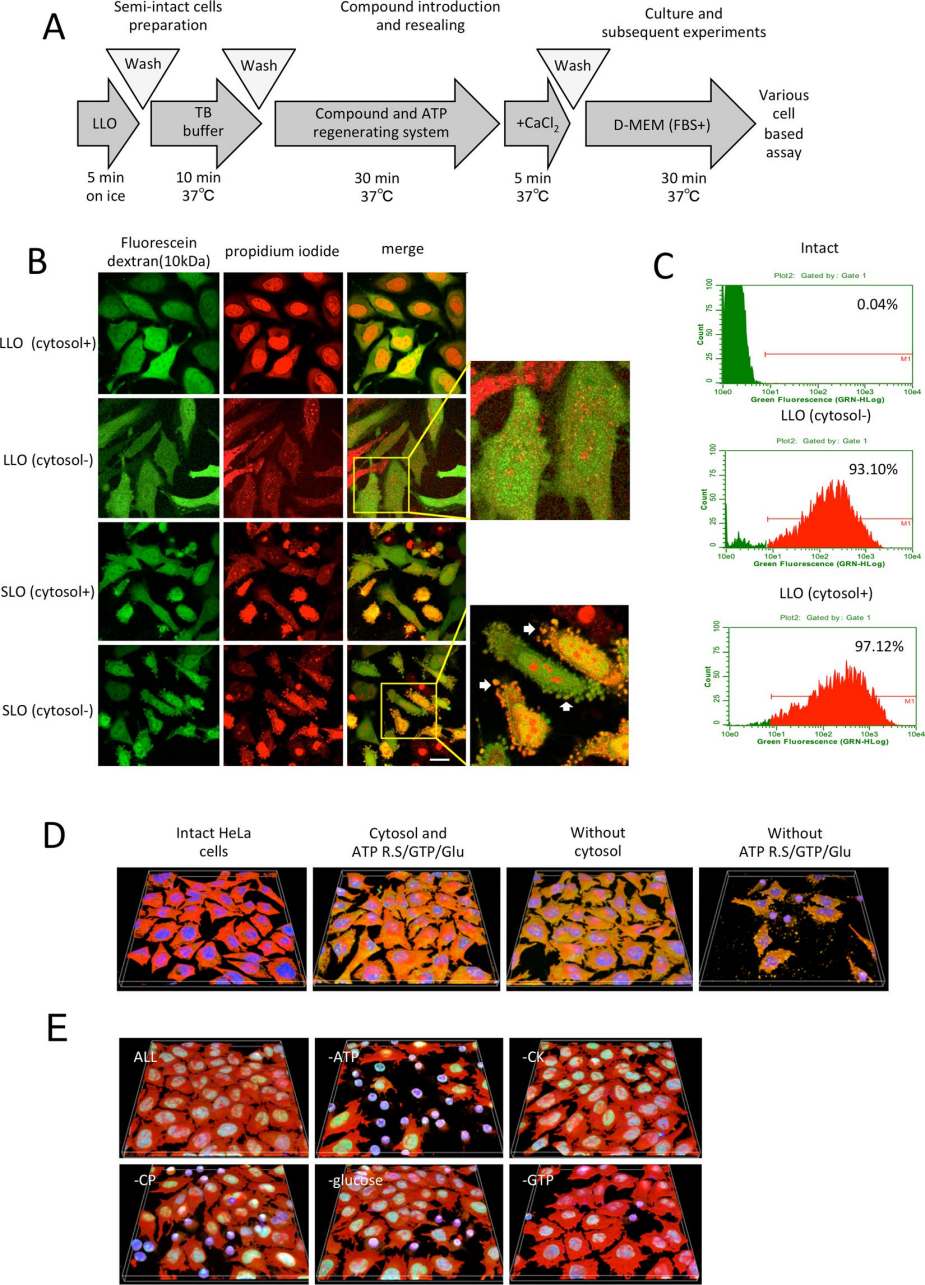
Cytosol-independent resealing of LLO-type semi-intact cells. (**A**) Basic protocol for LLO-type cell resealing. HeLa cells were permeabilized as described in Fig. 1A, and then were incubated with resealing buffer at 37 °C for 30 min. The cells were further incubated with 1 mM CaCl_2_ at 37 °C for 5 min to reseal the cells. After resealing, the cells were incubated with medium at 37 °C for more than 30 min. (**B**) HeLa cells were incubated with 0.15 μg/ml LLO or 0.125 μg/ml SLO on ice for 5 min. After washing with PBS, the cells were further incubated with prewarmed TB that contained propidium iodide at 37 °C for 10 min. The permeabilized cells were incubated with resealing buffer that contained 10 kDa dextran conjugated to fluorescein in the presence (cytosol+) or absence (cytosol−) of cytosol at 37 °C for 30 min. After resealing with 1 mM CaCl_2_ at 37 °C for 5 min, the cells were observed using confocal microscopy. Bar = 20 μm. (**C**) HeLa cells, treated as described in B, were subjected to flow cytometry. Intact cells were used as a negative control. The histograms of fluorescein fluorescence of dextran are shown. The percentage of fluorescein-positive resealed cells is also shown in the histogram. (**D**) Intact HeLa cells and resealed cells containing cytosol, ATP regenerating system, GTP and glucose (Cytosol and ATP R.S/GTP/Glu), ATP regenerating system, GTP and glucose (Without cytosol), or cytosol (Without ATP R.S/GTP/Glu) were prepared as described in Fig. S2. In the image, PI is yellow, tubulin is red, and DAPI is blue. The scan area is a square of side 317.44 μm on a side. (**E**) HeLa cells were permeabilized with LLO, and were incubated with resealing buffer (ALL) or resealing buffer except for ATP (−ATP), CK (−CK), CP (−CP), glucose (-glucose), or GTP (−GTP) at 37 °C for 30 min. After resealing, the cells were incubated with medium at 37 °C for 1 h and were subjected to immunofluorescence using antibodies against Lamin-A substrate (Green) and β-Tubulin (Red). The scan area is a square of side 317.44 μm on a side.

We next examined whether the LLO-mediated lesion of the HeLa cell plasma membrane is repaired by Ca^2+^ and cytosol as has been shown for the SLO-type resealing method. First, using the SLO-type resealing method as a reference (Fig. 2A), we examined the effect of calcium on repair of the membrane injury (henceforth referred to as “resealing”) in LLO-treated cells. LLO-mediated permeabilized HeLa cells were incubated with or without exogenous cytosol in the presence of an ATP regenerating system, GTP, glucose and 10 kDa fluorescently-labeled dextran (FL-dextran) for 30 min. After further incubation with 1 mM CaCl_2_ at 37 °C for 5 min, the cells were washed with medium (containing 3 mM CaCl_2_) and observed with confocal microscopy (Fig. 2B). As was observed during SLO-type resealing, retention of the fluorescent signal was confirmed in LLO-treated permebilized cells. This shows that LLO can be used for resealing. It has been reported that the LLO-mediated lesions at neutral pH allow passage of ions such as Ca^2+^ ^21,23,24^, but, here we observed passage of 10 kDa-dextran.

Interestingly, the resealing process was independent of cytosol as we observed retention of FL-dextran in the resealed cells without significant damage to cellular morphology in both the presence or absence of cytosol, whereas SLO treatment caused the substantial blebbing without cytosol (Fig. 2B). Additionally, flow cytometry analysis revealed that the percentage of cells containing FL-dextran was similar between cells resealed in the presence (97%) or absence (93%) of cytosol (Fig. 2C). We also confirmed the resealing of LLO-treated cells without cytosol in different cell types, mouse embryonic fibroblasts (MEF) and mouse methylcholanthrene-induced sarcoma cells (L-cell) (Fig. S1).

An ATP-regenerating system, GTP and glucose (ATP R.S/GTP/Glu) is necessary to maintain the shape of semi-intact cells produced by LLO, in which deficient integrity and dynamic regulation of actin filaments or microtubules might perturb cellular matrix adhesion. Absence of an ATP regenerating system, GTP and glucose during the resealing process led to cellular disruption and distorted morphology of remnant cells (Fig. 2D). Immunofluorescence analysis using anti-β-tubulin antibody also revealed that the disruption of the microtubules was dependent upon the ATP regenerating system but not cytosol (Fig. S2). In particular, exclusion of ATP from the ATP-regenerating system, GTP and glucose caused critical damage (Fig. 2E). Collectively, an ATP regenerating system, GTP and glucose is necessary for LLO-type resealing, probably because microtubule integrity, at least under our experimental conditions, was significantly perturbed in the absence of ATP^25^.

### Examination of cellular function of LLO-type resealed cell during the stress response, recycling of transferrin, and proliferation

We also examined the cellular stresses caused by permeabilization and resealing in the presence or absence of cytosol by monitoring the activation (or phosphorylation) of the stress response kinases, p38 MAPK or p42/44 MAPK, by Western blot analysis (Fig. S3). We detected transient activation of p38 MAPK and p42/44 MAPK 30 min after resealing, which declined after 1 h (Fig. S3). In addition, we observed no significant differences in the stress response in resealed cells prepared with or without cytosol, supporting the idea that LLO-type cell resealing requires no cytosol for resealing. Furthermore, to test the functional recovery of plasma membrane in LLO-type resealed cells, we examined the recycling process of fluorescently-labeled transferrin (Fig. S4). LLO-type resealed cells without cytosol was incubated with Alexa488-transferrin at 37 °C for 30 min, and then further with non-labeled transferrin in the presence or absence of monensin, an inhibitor of transferrin recycling by inhibiting acidification of endosomes via losing the proton gradient^26^. As shown in Fig. S5, the recycling of transferrin occurred, which was inhibited by monensin, indicating that the endocytic process including internalization, sorting, and recycling was restored in LLO-type resealed cells. We also found that the resealed cells proliferated sufficiently, and the proliferation of resealed cells appeared to be similar to that of intact cells (Fig. S5). These results indicate that LLO-type resealed cells are transiently stressed due to the permeabilization and resealing processes, but that after at least 1 h, cellular functions are restored sufficiently for cells to perform endocytosis and to proliferate.

### LLO-type resealing provides a new delivery system for introducing mid-weight molecules into cells

**Figure 3:**
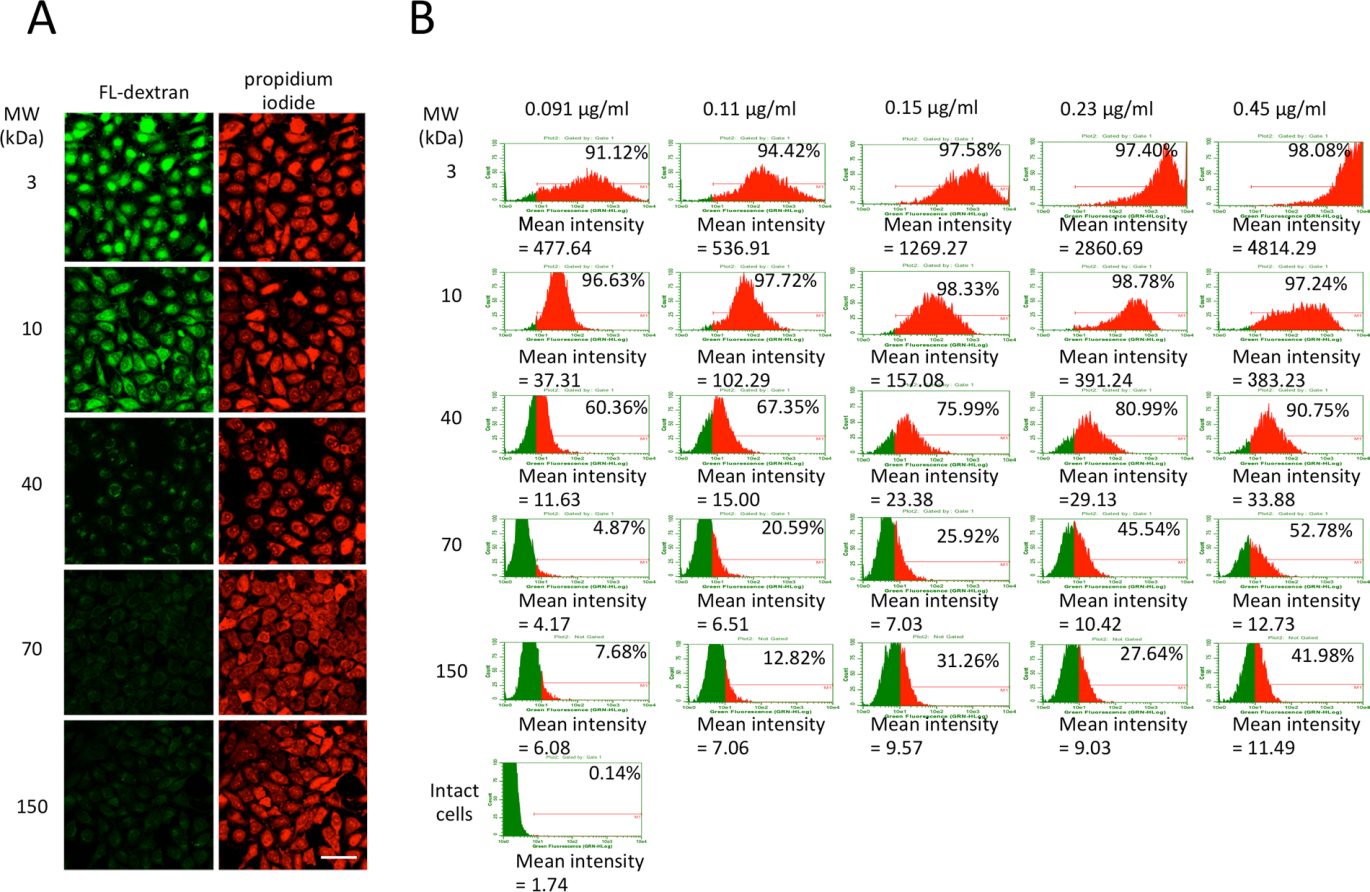
Evaluation of the molecular size of dextran that could be introduced into LLO-type resealed cells. (**A**) HeLa cells were permeabilized with 0.15 μg/ml LLO. The permeabilized cells were stained with propidium iodide and were further incubated with resealing buffer that contained fluorescently-labeled 3, 10, 40, 70 or 150 kDa dextran at 37 °C for 30 min. After resealing with CaCl_2_ treatment, the cells were incubated at 37 °C for 1 h with medium, and observed using confocal microscopy. Bar = 50 μm. (**B**) HeLa cells were permeabilized with 0.09, 0.11, 0.15, 0.23 or 0.45 μg/ml LLO and were incubated with resealing buffer that contained fluorescently-labeled 3, 10, 40, 70, or 150 kDa dextran at 37 °C for 30 min. The cells were resealed and subjected to flow cytometry. The histograms of fluorescein fluorescence of dextran are shown. The percentage of fluorescently-labeled dextran-positive cells and the mean fluorescence intensity of fluorescently-labeled dextran are also shown on the histograms.

We next examined the effect of molecular size upon the delivery of molecules by LLO-type resealing. LLO-type resealing of HeLa cells was prepared in the presence of PI and 3~150 kDa FL-dextran. After 1 h incubation in medium at 37 °C, the cells were subjected to confocal microscopy. We observed efficient delivery of 3 kDa or 10 kDa FL-dextran into cells by confocal microscopy. Little fluorescence of 40 kDa FL-dextran was observed, and no fluorescence of either 70 kDa or 150 kDa FL-dextran was observed in the resealed cells (Fig. 3A).

To evaluate the effect of LLO concentration on the size of molecules that can be delivered into cells, we treated HeLa cells with varying concentrations of LLO and incubated with 3~150 kDa FL-dextran. HeLa cells were treated with 0.091~0.45 µg/ml LLO and 3~150 kDa FL-dextran was introduced in the absence of cytosol. After resealing with calcium ion, the cells were incubated for 30 min and were analyzed by flow cytometry (Fig. 3B). This analysis revealed that 3 kDa dextran could enter cells at any concentration tested in this study and that the mean fluorescence intensity of intracellularly delivered 3 kDa FL-dextran increased in a LLO concentration-dependent manner. In contrast, with 10 kDa and 40 kDa dextran, there seemed to be an upper limit of the concentration of LLO, over which mean fluorescence intensity of introduced FL-dextran did not significantly increase in response to raising the concentration of LLO (0.23 µg/ml LLO for 10 kDa and 0. 15 µg/ml LLO for 40 kDa). Neither 70 kDa or 150 kDa dextran entered cells, as the mean fluorescence intensity remained at background levels even when cells were treated with LLO at high concentration (Fig. 3B).

We suppose that the average LLO-mediated lesions are of a size such that molecules <3 kDa can freely pass through the membrane, and its number increased depending on the LLO concentration. The large lesions through which 10 kDa or 40 kDa molecules could pass are generated at a mid-range of concentration of LLO. However, at higher concentrations, these large lesions do not appear to increase in number. The exact mechanisms behind this phenomenon remained unknown at this time, but we speculate that it might be due to structural hindrance of LLO oligomerization or the lack of free space on the membrane to dynamically interact with one another for creating large lesions at higher concentrations of LLO. These results suggest that the limit in size of molecules that can be introduced into cells can be controlled by the LLO concentration.

### Examination of outflow of intracellular cytosolic proteins from LLO-type resealed cells

**Figure 4:**
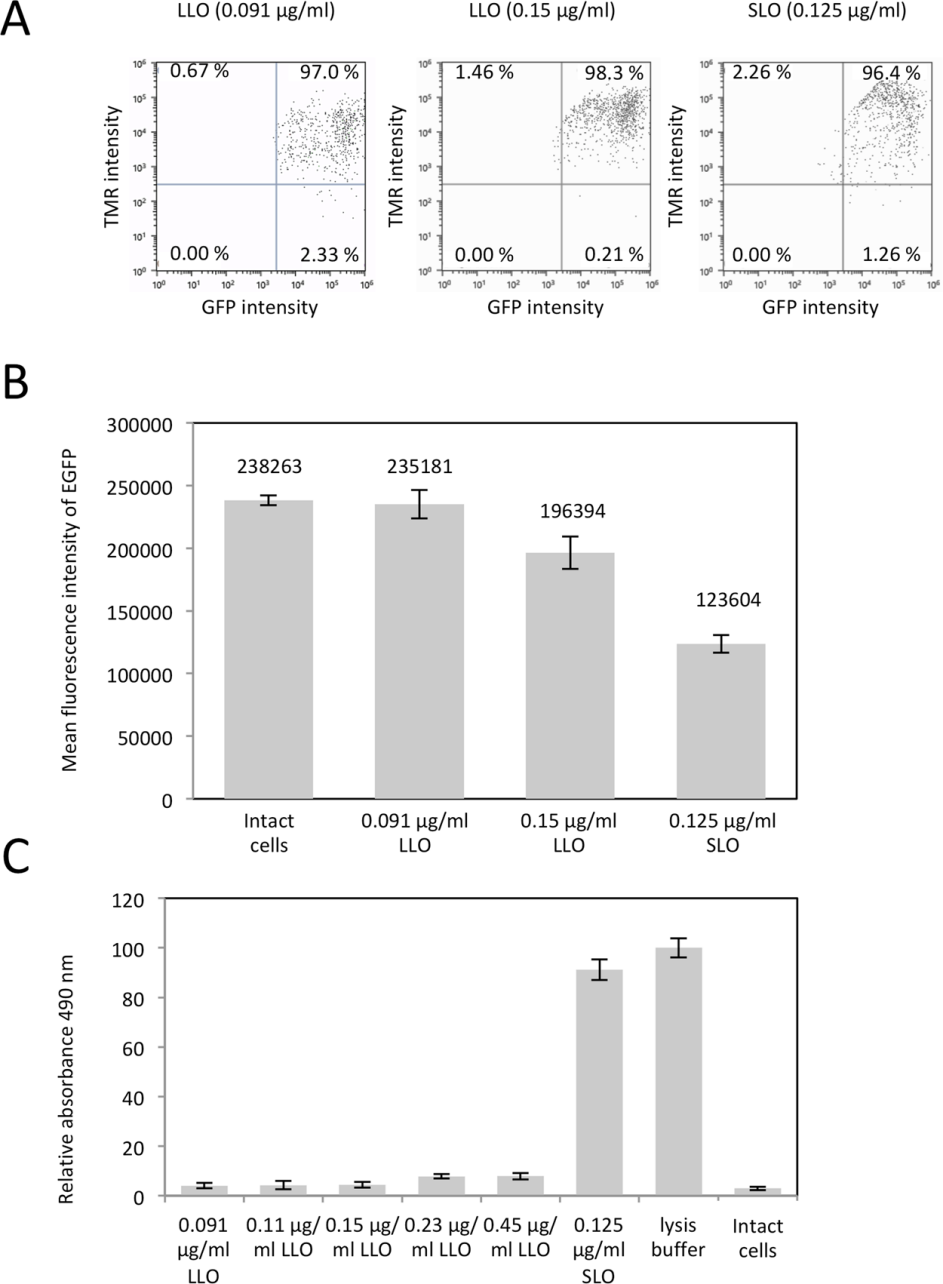
Evaluation of the outflow of cytosolic GFP and LDH from LLO-type resealed cells. HeLa cells stably expressing GFP were permeabilized with 0.091 or 0.15 μg/ml LLO or 0.125 μg/ml SLO as described in Fig. 1A, and were incubated with resealing buffer that contained 3 kDa dextran conjugated to TMR at 37 °C for 30 min. After resealing by addition of 1 mM CaCl_2_ for 5 min, the cells were incubated with medium for 30 min, and were tripsinized, and subjected to flow cytometry. The x- or y-axis indicates GFP or TMR fluorescence, respectively. The percentage of cells in each square are indicated on the plot. (**B**) The mean fluorescence intensity of GFP of intact cells or TMR-dextran positive resealed cells. Experiments were performed three times. Values are expressed as the mean ± standard deviation of each experiment. (**C**) HeLa cells were permeabilized with 0.091, 0.11, 0.15, 0.23, 0.45 µg/ml LLO or 0.125 µg/ml SLO and the supernatant was collected and was used to measure the activity of LDH, which leaked from the permeabilized cells. The mean ± standard deviation of relative absorbance normalized with buffer only (0%) and lysis buffer (100%) is shown in the graph. n = 3.

We next examined the outflow of cytosolic proteins from LLO-type resealed cells. Firstly, we used HeLa cells stably expressing GFP (HeLa-GFP), and examined the loss of GFP fluorescence during/after permeabilization by flow cytometry. HeLa-GFP cells were permeabilized with 0, 0.091, or 0.15 µg/ml LLO or 0.125 µg/ml SLO, and incubated with TB (LLO) or cytosol (SLO) in the presence of an ATP regenerating system, GTP, glucose, and TMR-dextran (3 kDa) at 37 °C for 30 min, and resealed. The fluorescence intensities of GFP and TMR-dextran in resealed cells were examined by flow cytometry (Fig. 4A,B). The percentage of cells exhibiting both GFP and TMR-dextran fluorescence was ~98% or ~96% in LLO-type or SLO-type resealed cells, respectively. The mean fluorescence intensity of GFP in the resealed cells treated with 0.091 µg/ml LLO (235,181) was similar to that observed in intact HeLa-GFP cells (238,263), whereas it decreased to 196,394 or 123,604 in the resealed cells treated with 0.15 µg/ml LLO or with 0.15 µg/ml SLO. Since treatment with 0.091 µg/ml LLO enabled the entry of 3 kDa dextran into cells (Fig. 3B, 0.091 µg/ml) but inhibited the leakage of intracellular GFP from the cells, the results suggest that the size of molecules that can be flow in or out from the cells can be controlled by varying the concentration of LLO.

We next examined whether molecules larger than GFP could be retained in the cells. We tested the leakage of lactate dehydrogenase (LDH), which is composed of 4 subunits with a molecular size of 140 kDa, from LLO-treated cells. HeLa cells were treated with LLO at varying concentrations 0.091~0.45 µg/ml or 0.125 μg/ml SLO, and the activity of LDH, which leaked into the supernatant was measured by LDH assay. The leakage of LDH was substantially repressed in cells treated with LLO at all concentrations tested in this study (Fig. 4C). In contrast, SLO treatment induced 91.5% leakage of LDH from the cells (Fig. 4C). This indicates that large molecules such as LDH are completely retained in LLO-type resealed cells.

In addition, the total protein prepared from LLO-type permeabilized cells or from intact cells was compared by Coomassie brilliant blue (CBB) staining. HeLa cells were incubated with or without LLO on ice for 5 min. After incubation at 37 °C for 10 min, the cells were lysed and total protein was separated by SDS-PAGE and stained by CBB. As shown in Fig. S6, no significant difference in the banding pattern was observed, indicating that the composition of the proteins appeared similar.

### Semi-quantification of the intracellular concentration and retention of molecules introduced into LLO-type resealed cells

**Figure 5:**
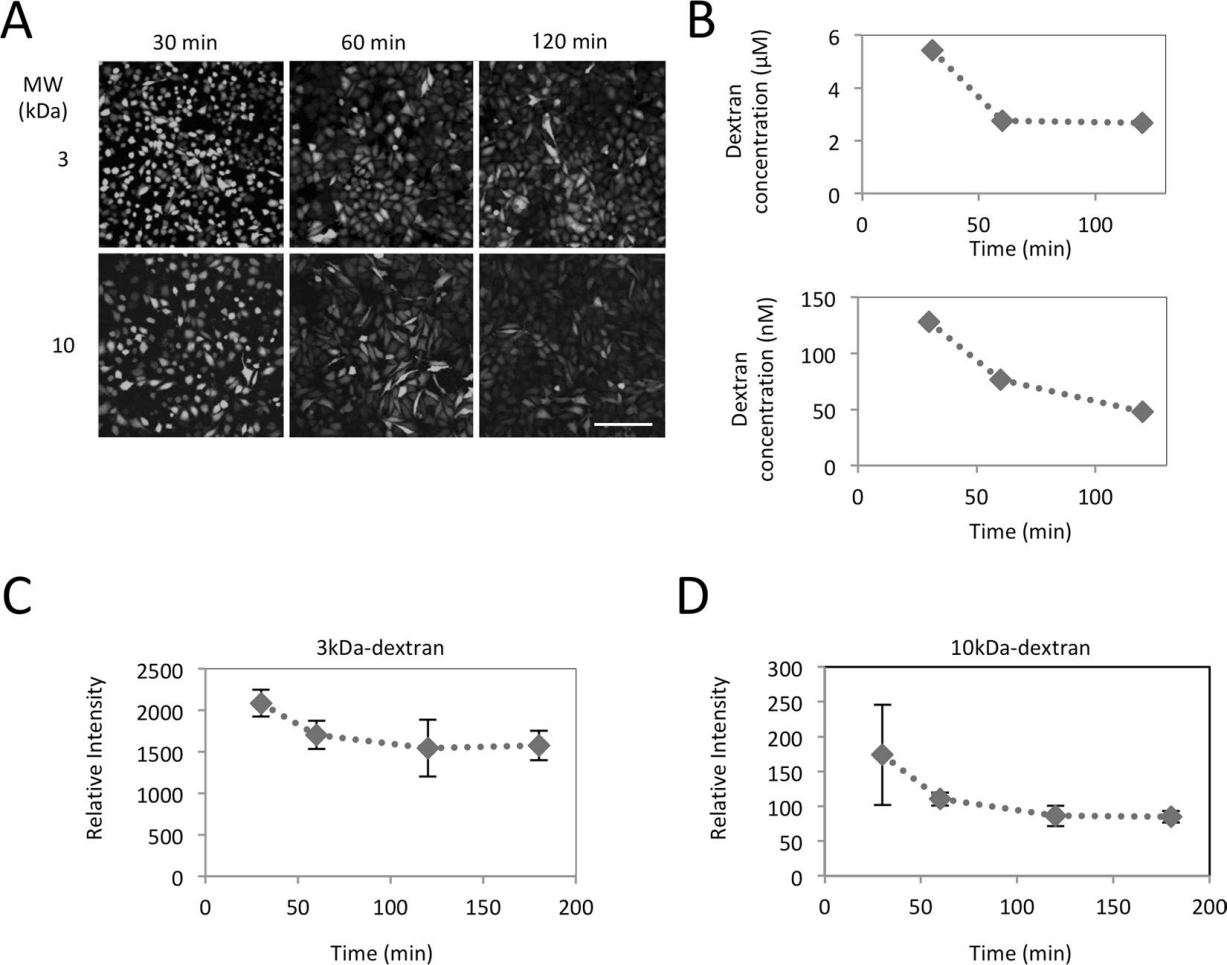
Evaluation of the intracellular concentration of fluorescein-dextran in LLO-type resealed cells. (**A**) HeLa cells were permeabilized with LLO, and were incubated with resealing buffer that contained 300 μg/ml 3 or 10 kDa dextran conjugated with fluorescein. After resealing, the cells were incubated at 37 °C for 30, 60 or 90 min with medium, and observed using confocal microscopy. Bar = 200 µm. (**B**) Mean fluorescence intensities of fluorescein in the intracellular area were calculated from the image obtained in (**A**), and the intracellular concentration of the dextran was estimated from the standard curve. (**C**,**D**) As with A, 3 kDa (**C**) or 10 kDa (**D**) fluorescently labeled dextran was introduced into HeLa cells. After resealing, the cells incubated with medium for 30, 60, 90, 120 or 180 mim and subjected to flow cytometry. Experiments were performed three times. The mean ± standard deviation mean of fluorescence intensity is shown in the graph.

We next estimated the intracellular concentrations of introduced molecules in resealed HeLa cells using semi-quantitative fluorescence image analysis. After introducing FL-dextran into cells by LLO-type resealing, fluorescent images of 1.86 µm thickness were obtained by confocal microscopy at 30, 60, and 120 min after resealing. As diffuse fluorescence of soluble FL-dextran was observed in each cell, the mean pixel intensity of the fluorescence image appeared to represent the concentration of FL-dextran in the resealed cell (Fig. 5A,B). A calibration curve was created by using serial dilutions of a solution of 3 kDa or 10 kDa FL-dextran. When LLO-treated, semi-intact HeLa cells were incubated with 100 µM FL-dextran (3 kDa) and resealed, the intracellular concentration of 3 kDa dextran was ~5.8 µM at 30 min, and this was reduced by half after 60 min. For 10 kDa FL-dextran, the intracellular concentration was ~130 nM after 30 min, which was 1/285 of the initial concentration in the mixture (30 µM). This result is consistent with the flow cytometry analysis in Fig. 3, indicating the favorable delivery of small-size molecules in LLO-type resealed cells.

We next examined the retention time of introduced molecules in LLO-type resealed cells. After introduction of 3 kDa or 10 kDa fluorescein-dextran by LLO-type cell resealing, the cells were incubated with medium at 37 °C, 5% CO_2_ for 30, 60, 120, and 180 min, and were subjected to flow cytometry (Fig. 5C,D). We found that the mean fluorescence of intracellular 3 kDa (Fig. 5C) and 10 kDa (Fig. 5D) dextran was gradually decreased until 60 min, which might be caused by the incomplete repair of the injured cell membrane or the washout of the extracellular dextran, which bound non-specifically to the cell membrane. However, the decrease of intracellular dextran subsequently stopped, and was nearly unchanged at 120 or 180 min. These results suggest that, although the introduced molecules might leak during a 60 min period after resealing, the introduced molecules are stably retained in LLO-type resealed cells thereafter.

We supposed that it might be important to assay cellular function at times >60 min after resealing under this experimental condition, because times greater than 60 min would be necessary to restore both the retention activity of introduced molecules and recovery from stress (Fig. S3). The time required for membrane repair might be reduced at low concentrations of LLO, which remains to be elucidated in future studies. Additionally, screening of drugs that prevent leakage or that promote repair of the injured membrane should be effective for making LLO-type resealing more suitable for cell-based analyses.

### *In vivo* functional analysis of membrane-impermeable low-molecular weight molecule by LLO-type resealing

One of the aims of this study is to evaluate the intracellular activity of delivered biomolecule in resealed cells. We next examined the intracellular activation of protein kinase A (PKA) by cAMP or its membrane-impermeable/permeable analogues. We first investigated the phosphorylation of PKA substrate protein by the membrane permeable cAMP analogue, db-cAMP, to find suitable substrate proteins that could serve as a sensitive indicator for PKA activation. HeLa cells were treated with db-cAMP (Mw = 491.4) or H89, a membrane permeable inhibitor of PKA, at varying concentrations for 60 min. The cells were lysed and subjected to Western blotting using anti-phospho- (Ser/Thr) PKA substrate antibody. As shown in Fig. S7, we detected nine polypeptide bands that were phosphorylated in the presence of db-cAMP but not of H89. Band e, one of the polypeptide bands that responded to db-cAMP treatment as above, was chosen as a sensitive indicator for quantitative PKA activation, although we were unable to identify this polypeptide band.

**Figure 6:**
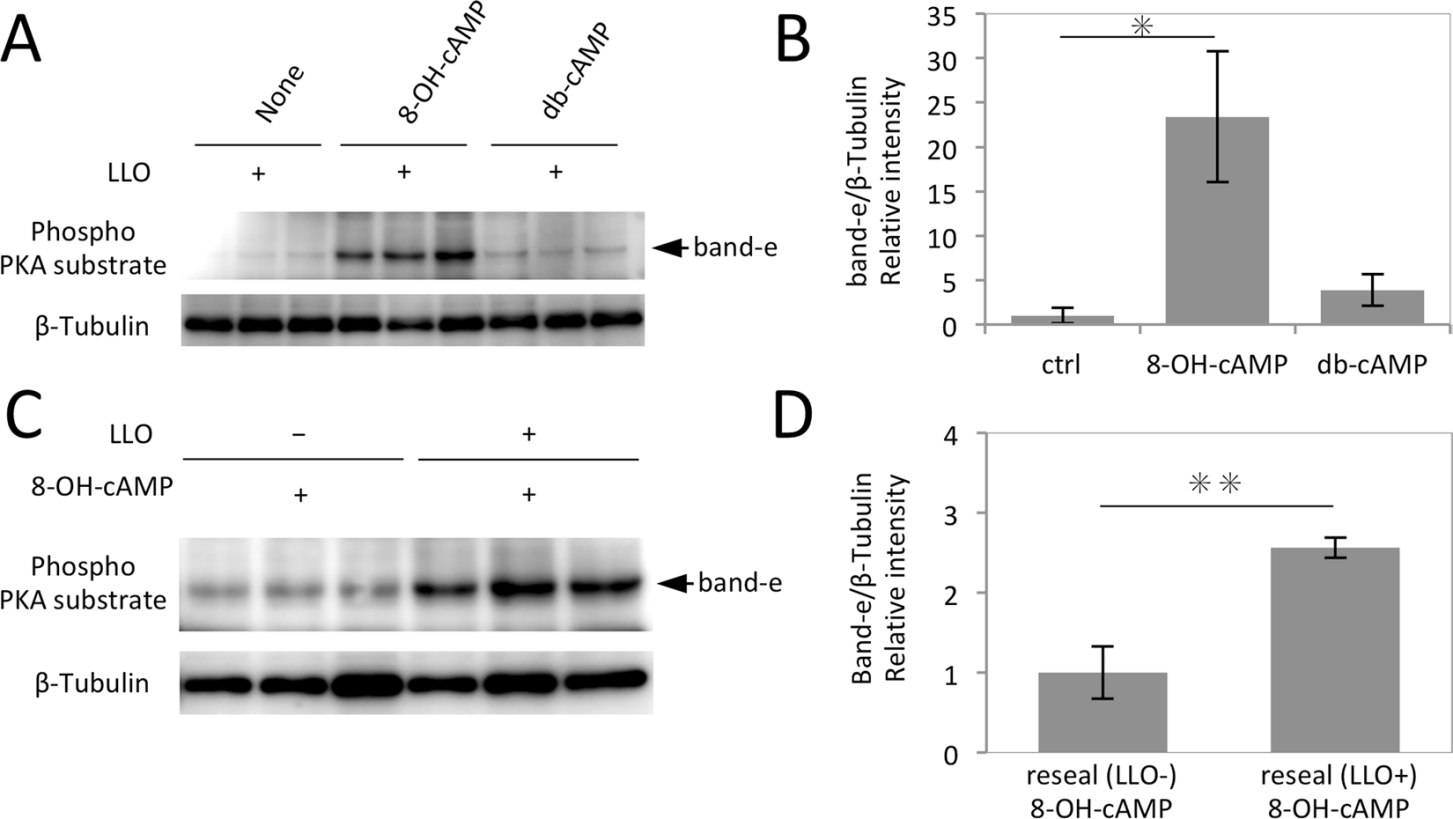
Activation of PKA by introducing membrane-impermeable cAMP analogues by LLO-type resealing. (**A**) HeLa cells were permeabilized with 0.15 μg/ml LLO, and were incubated with resealing buffer in the presence or absence (None) of 1 mM 8-OH-cAMP or 1 mM db-cAMP at 37 °C for 30 min. After resealing, the cells were incubated with medium at 37 °C for 1 h and were subjected to Western blotting using antibodies against Phospho-PKA substrate and β-Tublin. The arrow indicates band e. (**B**) The intensities of band e and β-Tublin in (**A**) were calculated and the means and the standard deviations of the relative intensity of band e to β-Tublin are shown in the graph. The mean intensity of LLO-type resealed cells with no components (None in (**A**)) was set to 1. n = 3. *P value =  < 0.05. (**C**) HeLa cells were incubated with (LLO+) or without (LLO -) LLO, and were incubated with TB 37 °C for 10 min. The cells were incubated with resealing buffer that contained 1 mM 8-OH-cAMP at 37 °C for 30 min. After resealing, the cells were incubated with medium at 37 °C for 1 h and were subjected to Western blotting using antibodies against Phospho-PKA substrate and β-Tublin. The arrow indicates band e. (**D**) The intensities of band e and β-Tublin in (**C**) were calculated and the means and the standard deviations of the relative intensity of band e to β-Tublin are shown in the graph. The relative intensity of the cells without LLO treatment was set to 1. *P* value was calculated using paired student’s t-test. n = 3. **P value =  < 0.01.

Next, using the same experimental procedure, we examined the effect of the membrane impermeable cAMP analogue, 8-OH-cAMP (MW = 367.2)^27^, on PKA activation in LLO-type resealed cells. LLO-mediated permeabilized HeLa cells were incubated with 1 mM 8-OH-cAMP or 1 mM db-cAMP for 30 min and resealed. Then, the cells were incubated for another 60 min with medium and lysed. WB analysis revealed that the intensity of band e was significantly increased by 8-OH-cAMP, indicating that introduction of 8-OH-cAMP into LLO-type resealed HeLa cells successfully activated PKA (Fig. 6A,B). We also confirmed that the activation was dependent upon LLO (Fig. 6C,D). Interestingly, the membrane permeable cAMP analogue, db-cAMP, activated PKA to a lesser extent than 8-OH-cAMP (Fig. 6A,B). We suppose that intracellularily delivered db-cAMP might diffuse from the cells through the plasma membrane during incubation owing to its membrane permeability. As such, LLO-type resealing might prove useful for evaluating the intracellular retention of various introduced compounds, which could be beneficial for understanding the efficacy and the side effect of drugs. Furthermore, we performed the similar experiments using murine lymphoma EL4 cells, confirming that this method was also applicable for suspended cell systems (Fig. S8).

### Membrane-impermeable Akt-in peptide with mid-sized molecular weight inhibited EGF-triggered activation of Akt in LLO-type resealed cells

**Figure 7:**
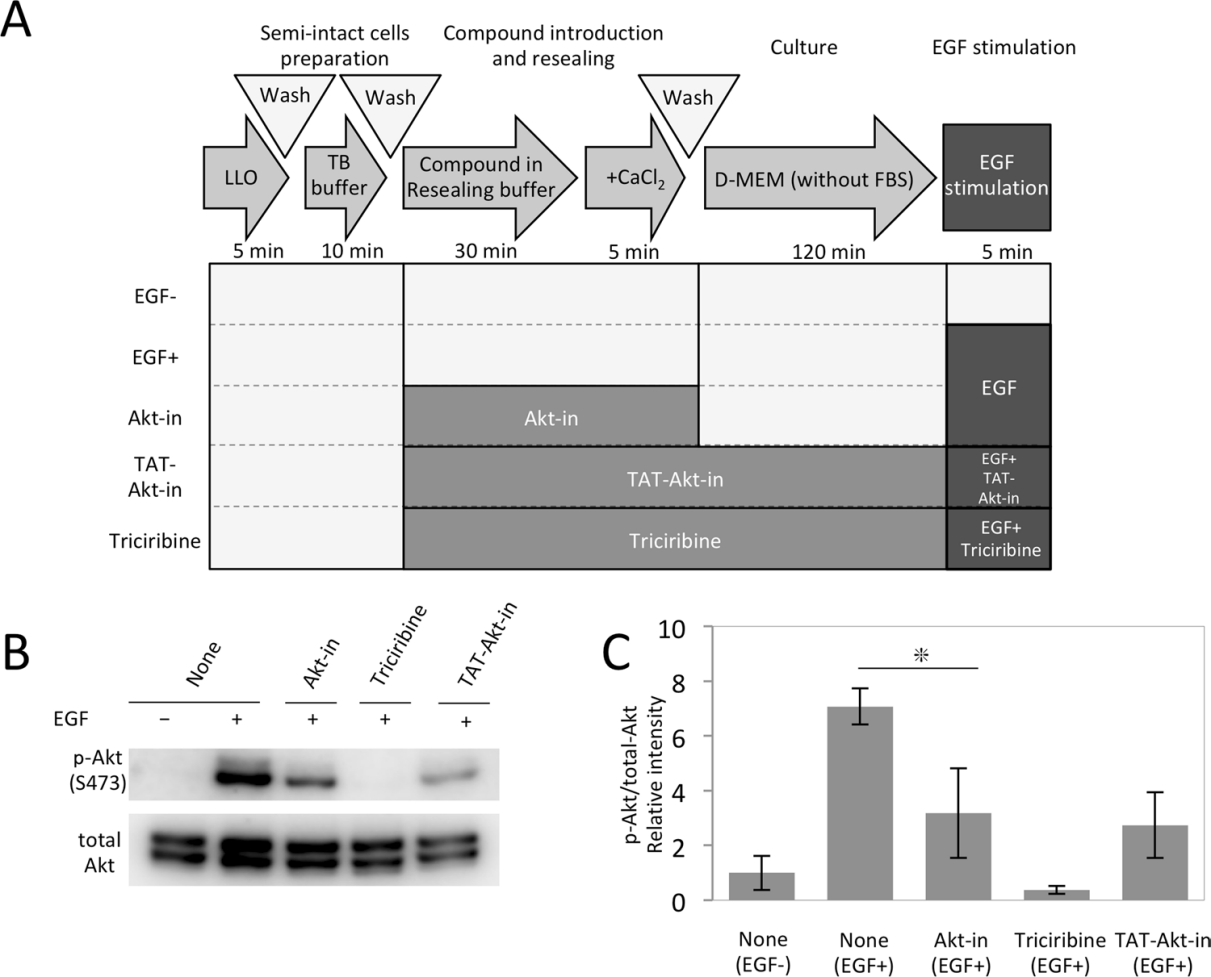
Activation of Akt by membrane impermeable peptide, Akt-in, by LLO-type cell resealing. (**A**) Scheme of the evaluation of activity of membrane-impermeable peptide by LLO-type cell resealing. HeLa cells were permeabilized with LLO, and were incubated with resealing buffer with or without 1 mM Akt-in, 50 μM TAT-Akt-in, or 10 μg/ml Triciribine at 37 °C for 30 min. After resealing by addition of CaCl_2_, the cells were incubated with serum free medium at 37 °C for 2 h. When the cells were exposed to TAT-Akt-in or Triciribine, these molecules were also included in the serum-free medium. After incubation, the cells were stimulated with or without 10 ng/ml EGF in serum free medium at 37 °C for 5 min. (**B**) HeLa cells were treated as described in (**A**), lysed, and were subjected to Western blotting using antibodies against phosphorylated Akt at S473 (p-AKT S473) and pan-Akt (total-Akt). (**C**) The band intensities in (**B**) were calculated, and the means and the standard deviations of the relative band intensity of phosphorylated Akt to total Akt are shown in the graph. The relative intensity in LLO-type resealed cells without EGF treatment was set to 1. n = 3. *P value =  < 0.05.

We next examined the intracellular function of the Akt-inhibitor VI (Akt-in), using the LLO-type resealing method. Akt-in is a membrane-impermeable peptide comprising 15 amino acids. TAT-conjugated Akt-in (TAT-Akt-in) was synthesized as a membrane-permeable inhibitor of phosphorylation of Akt at S473 (Akt activation) in HEK293 cells^28^. The effect of Akt-in was evaluated by monitoring the phosphorylation of Akt at S473 by WB analysis of cell lysates. Several different types of cell lysate were prepared as shown in Fig. 7A: (control:−EGF and +EGF) LLO-permeabilized HeLa cells were exposed only to resealing buffer and the resulting cells were incubated with DMEM without serum and stimulated without (-EGF) or with(+EGF) EGF for 5 min. (Akt-in) resealed cells were prepared and the EGF stimulation assay was performed similarly to the control but the resealing solution contained 1 mM Akt-in. (membrane permeable inhibitor: Triciribine and TAT-Akt-in) the resealed cells were prepared according to the same procedure as the control and subjected to the EGF stimulation assay, but the resealing solution and the following assay solution also contained 10 μg/ml Tricibirine or 50 μM TAT-Akt-in. For the cell lysates prepared as above, WB analysis was performed using antibodies against phosphorylated Akt (p-AKT S473) or pan-Akt (total Akt). For the (control) samples, an EGF-stimulated increase in phosphorylated Akt was observed (compare −EGF and +EGF in Fig. 7B). Membrane-permeable and low-molecular weight inhibitor (Triciribine) and mid-sized inhibitory peptide (TAT-Akt-in) repressed Akt phosphorylation substantially (Triciribine and TAT-AKT-in in Fig. 7B,C). Interestingly, Akt-in showed a similar inhibitory effect as membrane-permeable TAT-Akt-in in this experiment (compare Akt-in to TAT-AKT-in in Fig. 7B,C). According to the estimation as shown in Fig. 5, the final intracellular concentration of Akt-in in cells might be ~50 µM, which is roughly the same concentration of TAT-Akt-in in cells. This result indicates that our LLO-type resealing method enables the analysis of intracellular function of membrane-impermeable Akt-in peptide without modification with any modification, which is beneficial not only because of eliminating the time- and labor-consuming process of modification, but also because of avoiding the potentially confounding effects caused by addition of the extra modification.

Functional peptides are promising biopharmaceutical cargos to deliver using micelles or liposomes in the body. In such cases, membrane impermeability is rather favorable since the agents are retained in micelles or liposomes until they reach and fuse to the target cells or tissues. As such, the need for a system to evaluate the intracellular effectiveness of membrane-impermeable peptides should increase in the near future.

SLO-type and LLO-type resealing have different advantages and the method for cell resealing should be chosen according to the purpose of the experiments. Firstly, LLO-type resealing excels in terms of a lack of requirement for cytosol in the resealing process. LLO-type resealing occurs independent of cytosol (Fig. 2C) and LLO-type resealed cells are morphologically intact even without cytosol (Fig. 2D). In contrast, without cytosol, blebs are extensively formed in SLO-treated resealed cells and most cells die (Fig. 2B). Secondly, LLO-type resealing is preferable to introduce low- and mid-sized molecules into cells. No exchange of large molecules in LLO-treated cells was demonstrated in Figs 3 and 4. In addition, we examined leakage of lactate dehydrogenase (LDH), a cytosolic protein which forms a tetramer (~140 kDa), and found that only background levels of LDH leaked following LLO treatment whereas there was ~90% leakage of LDH from SLO-treated cells (Fig. 4C). Furthermore, we also demonstrated that GFP, which is 27 kDa, did not flow out from the cells when the cells were permeabilized with LLO at low concentration (<0.091 μg/ml) (Fig. 4B). We confirmed the utility of LLO-type resealing in various cell types such as mouse embryonic fibroblasts (MEF), mouse methylcholanthrene-induced sarcoma cells (L-cell), and murine lymphoma-derived, non-adherent cells (EL4). So LLO-type resealing is more useful for specifically introducing small or mid-sized molecules into cells without the requirement for cytosol. This advantage of LLO-type resealing should facilitate high throughput screening of membrane-impermeable small chemical compounds to test their intracellular activities. Candidate membrane-impermeable compounds, identified by cell-based assays using LLO-type resealed cells, could be further modified for pharmaceutical use, such as by conjugating to CPP peptide to generate membrane permeability. So, LLO-type resealing could be used as support system for discovering novel drugs that have been ignored due to their membrane impermeability, yet are still effective as medicine in cells.

In contrast, SLO-type cell resealing is suitable for the complete exchange of cytosol representing different states since it allows both outflow and inflow of molecules of various sizes into cells. For example, the reconstitution of biological processes by replacement of cytosol, such as the reconstitution of cell-cycle dependent morphological changes of organelles by mitotic cytosol, can be achieved only by SLO-type cell resealing. So, one must choose the types of resealing method according to the aims of the experiment.

LLO-type resealing is suitable for assaying the intracellular function of low- or mid-size molecules. These molecules include not only biopharmaceutical products such as peptides but also the metabolites and intermediate products. So, this system is also applicable to study cellular metabolic pathways *in situ*. Collectively, our LLO-type resealing methods comprise complementary techniques for introducing membrane-impermeable biomolecules of various sizes into cells, and provide a novel ‘editing system’ of cellular functions in the near future.

## Materials and Methods

### Reagents and antibodies

Dextran (3, 10, 40, 70 kDa) conjugated to fluorescein or TMR was purchased from Invitrogen. Dextran (150 kDa) conjugated to FITC was purchased from TdB. Db-cAMP and 8-OH-cAMP were purchased from Biolog. H89 was purchased from Cayman Chemical. Akt-in and Triciribine were purchased from Calbiochem. ATP, GTP, Creatine kinase and Creatine phosphate were purchased from SIGMA. Glucose was purchased from Wako. Propidium iodide was purchased from Molecular Probes. The following primary antibodies were used: Rabbit anti-Phospho-(Ser/Thr) PKA Substrate antibody (CST 9621); Mouse GAPDH antibody (Millipore MAB374); Mouse anti-β Tubulin antibody (SIGMA T8328); Rabbit anti-Akt antibody (CST 4691); Rabbit anti-Phosphorylated Akt (S473) antibody (CST 4060); Rabbit anti-p42/44 MAPK antibody (CST4695), Rabbit anti-Phospho-p42/44 antibody (CST4370), Rabbit anti-p38 antibody (CST 9212) and Rabbit anti-Phospho-p38 antibody (CST 4511). The following secondary antibodies were used: Anti-Rabbit IgG, HRP-linked Antibody (CST 7074); Anti- Mouse IgG, HRP-linked Antibody (Promega W402B).

### Cell culture

HeLa cells were cultured in DMEM (Nissui) supplemented with 10% fetal bovine serum (SIGMA) and 1% penicillin/streptomycin (GIBCO). HeLa-GFP cells, which continuously express GFP, were grown in the same medium with the addition of 500 µg/ml Geneticin (GIBCO).

### Basic method of SLO-type resealing and preparation of cytosol

SLO-type resealing method was performed as described in Kano *et al*.^14^. Cytosol from murine lymphoma L5178Y cells was prepared as described in Kano *et al*.^29^.

### Preparation of LLO-mediated permeabilized cells

HeLa cells were grown on glass-bottomed dishes (Iwaki) or cover glasses (Matsunami). The cells were washed twice with PBS and then incubated with listeriolysisn O (LLO; Cedarlane) at the concentrations indicated in the text for 5 min on ice. After washing three times with Transport Buffer (TB: 25 mM Hepes-KOH, 115 mM potassium acetate, 25 mM MgCl2, pH7.4, and 2 mM EGTA), cells were incubated with TB containing 300 µg/ml PI and then washed twice with TB. The cells were fixed with 4% paraformaldehyde at room temperature for 20 min. After washing three times with PBS, the cells were observed using an LSM510 confocal microscope (Carl Zeiss).

### Resealing of LLO-mediated permeabilized cells

LLO-mediated permeabilized HeLa cells were incubated with resealing buffer (1 mM ATP, 8 mM creatine kinase and 50 μg/ml creatine phosphate, 1 mg/ml glucose, 1 mM GTP, 25 mM Hepes-KOH, 115 mM potassium acetate, 25 mM MgCl_2_, pH7.4) that contained the molecules indicated in the text at 37 °C for 30 min. Then, 1 mM CaCl_2_ was added and the cells were incubated at 37 °C for 5 min. The cells were washed twice with medium and were further incubated with pre-warmed medium at 37 °C, 5% CO_2_ for more than 30 min. The cells were washed twice with PBS, and were observed by using an LSM510 confocal microscope or were subjected to flow cytometry using Guava easyCyte HT flow cytometry system (Millipore).

### Measurement of intracellular protein retention

HeLa cells or HeLa cells stably expressing GFP were grown on cover glasses. The cells were washed twice with PBS and then incubated with 0, 0.09, 0.15 µg/ml LLO or 0.125 μg/ml SLO for 5 min on ice. After washing three times with TB, the cells were incubated with prewarmed TB at 37 °C for 10 min. The permeabilized cells were incubated with resealing buffer that contained 3 kDa dextran conjugated to tetramethyl rhodamine (TMR) at 37 °C for 30 min. Then 1 mM CaCl_2_ was added and the cells were incubated at 37 °C for 5 min. The cells were washed twice with medium and were further incubated with prewarmed medium at 37 °C, 5% CO_2_ for 30 min. The cells were trypsinized and subjected to flow cytometry using a SH800 cell sorter (Sony).

### Estimation of the concentration of fluorescein-conjugated dextran in resealed cells

LLO-mediated permeabilized HeLa cells were incubated with resealing buffer that contained 300 µg/ml of fluorescein-conjugated 3 or 10 kDa dextran at 37 °C for 30 min. Then 1 mM CaCl_2_ was added and the cells were incubated at 37 °C for 5 min. The cells were washed twice with medium and were further incubated with pre-warmed medium at 37 °C, 5% CO_2_ for 30, 60 or 120 min. After washing with PBS twice, the cells were observed under a Nikon A1 confocal microscope. NIS elements software (Nikon) was used to calculate the average intensity of fluorescein-conjugated dextran in the cell area. The concentration of intracellular dextran was estimated using a standard curve prepared using a solution containing 0~100 μM dextran (3, 10 kDa) conjugated with fluorescein.

### Lactate dehydrogenase (LDH) assay

HeLa cells were treated with or without SLO, LLO at the concentration indicated in the text on ice for 5 min, washed with ice-cold PBS three times, and incubated with TB at 37 °C for 10 min. The supernatant was collected and LDH activity in the supernatant was determined using cytotoxicity LDH assay kit-WST (Dojindo) according to the manufacturer’s instructions.

### Western blotting

Cells were lysed and separated by SDS-PAGE, and were transferred to PVDF membrane using a semi-dry blotting system at 12 V 60 min (Bio-Rad). After blocking with 5% BSA and 0.1% Tween20 in TBS, the membrane was incubated with primary antibody at 4 °C overnight, and then further with HRP-conjugated secondary antibody at room temperature for 1 h. The proteins were detected by enhanced chemiluminescence (Western Lightning Plus-ECL, PerkinElmer). The intensity of the bands was quantified using image-J.

### Measurement of cAMP analog activity

LLO-mediated permeabilized HeLa cells were incubated at 37 °C for 30 min with resealing buffer in the presence or absence of 1 mM 8-OH-cAMP or 1 mM db-cAMP. After resealing by addition of 1 mM CaCl_2_ at 37 °C for 5 min, the cells were washed twice with medium and were further incubated with pre-warmed medium at 37 °C, 5% CO_2_ for 60 min. The cells were lysed and Western blotting was performed using anti-phospho PKA substrate antibody and anti-β-tubulin antibody.

### Measurement of Akt-in activity

LLO-mediated permeabilized HeLa cells were incubated with resealing buffer in the presence or absence of 1 mM Akt-in, 50 µM TAT-Akt-in, or 10 µg/ml Triciribine at 37 °C for 30 min. After resealing with 1 mM CaCl_2_ at 37 °C for 5 min, the cells were washed twice with medium and were further incubated with pre-warmed medium with or without 50 µM TAT-Akt-in, 10 µg/ml Triciribine at 37 °C, 5% CO_2_ for 60 min. The cells were lysed and subjected to Western blotting using anti-phospho Akt antibody and anti-Akt antibody as primary antibody.

### Statistical test

Data analysis was carried out using the F-test to check the equality of variance and then the Student’s or Welch’s t-test. Experiments were performed three times. Values are expressed as the mean ± standard deviation (SD), and data were considered significant at *P < 0.05, **P < 0.01.

## Electronic supplementary material

Supplementary Information

## References

[1] O’Connor TP, Crystal RG (2006). Genetic medicines: treatment strategies for hereditary disorders. Nat. Rev. Genet..

[2] Ståhl S (2017). Affibody Molecules in Biotechnological and Medical Applications. Trends Biotechnol..

[3] Schuurman J, Graus YF, Labrijn AF, Ruuls SR, Parren PW (2014). Opening the door to innovation. MAbs.

[4] Kawamura A (2017). Highly selective inhibition of histone demethylases by de novo macrocyclic peptides. Nat. Commun..

[5] Zimmerman SB, Trach SO (1991). Estimation of macromolecule concentrations and excluded volume effects for the cytoplasm of Escherichia coli. J. Mol. Biol..

[6] Sarkar M, Smith AE, Pielak GJ (2013). Impact of reconstituted cytosol on protein stability. Proc. Natl. Acad. Sci..

[7] Sadaie W, Harada Y, Matsuda M, Aoki K (2014). Quantitative *In Vivo* Fluorescence Cross-Correlation Analyses Highlight the Importance of Competitive Effects in the Regulation of Protein-Protein Interactions. Mol. Cell. Biol..

[8] Said Hassane F, Saleh AF, Abes R, Gait MJ, Lebleu B (2010). Cell penetrating peptides: Overview and applications to the delivery of oligonucleotides. Cell. Mol. Life Sci..

[9] Kunisawa J, Nakagawa S, Mayumi T (2001). Pharmacotherapy by intracellular delivery of drugs using fusogenic liposomes: Application to vaccine development. Adv. Drug Deliv. Rev..

[10] Kreis TE (1986). Microinjected antibodies against the cytoplasmic domain of vesicular stomatitis virus glycoprotein block its transport to the cell surface. EMBO J..

[11] Bommert K (1993). Inhibition of Neurotransmitter release by C2-domain peptide impicates synaptotagmin in exocytosis. Nature.

[12] Schönenberger C, Schütz A, Franco-Obregón A, Zenobi-Wong M (2011). Efficient electroporation of peptides into adherent cells: Investigation of the role of mechano-growth factor in chondrocyte culture. Biotechnol. Lett..

[13] Schwarze, S. R. Biologically Active Protein into the Mouse In Vivo Protein Transduction: *Delivery of a Biologically Active Protein into the Mouse*. **1569**, (2009). 10.1126/science.285.5433.1569 10477521

[14] Kano F, Nakatsu D, Noguchi Y, Yamamoto A, Murata M (2012). A Resealed-Cell System for Analyzing Pathogenic Intracellular Events: Perturbation of Endocytic Pathways under Diabetic Conditions. PLoS One.

[15] Walch M, Ziegler U, Groscurth P (2000). Effect of streptolysin O on the microelasticity of human platelets analyzed by atomic force microscopy. Ultramicroscopy.

[16] Walev I (2001). Delivery of proteins into living cells by reversible membrane permeabilization with streptolysin-O. Proc. Natl. Acad. Sci. USA.

[17] Idone V (2008). Repair of injured plasma membrane by rapid Ca2+ dependent endocytosis. J. Cell Biol..

[18] Keyel PA (2011). Streptolysin O clearance through sequestration into blebs that bud passively from the plasma membrane. J. Cell Sci..

[19] Köster S (2014). Crystal structure of listeriolysin O reveals molecular details of oligomerization and pore formation. Nat. Commun..

[20] Schuerch DW, Wilson-Kubalek EM, Tweten RK (2005). Molecular basis of listeriolysin O pH dependence. Proc. Natl. Acad. Sci..

[21] Repp H (2002). Listeriolysin of Listeria monocytogenes forms Ca2+-permeable pores leading to intracellular Ca2+ oscillations. Cell. Microbiol..

[22] Hamon M, Bierne H, Cossart P (2006). Listeria monocytogenes: a multifaceted model. Nat. Rev. Microbiol..

[23] Vadia S, Seveau S (2014). Fluxes of Ca2+ and K+ are required for the listeriolysin O-dependent internalization pathway of Listeria monocytogenes. Infect. Immun..

[24] Vadia S (2011). The pore-forming toxin listeriolysin o mediates a novel entry pathway of L. monocytogenes into human hepatocytes. PLoS Pathog..

[25] Bershadsky AD, Gelfand VI (1981). ATP-dependent regulation of cytoplasmic microtubule disassembly. Proc. Natl. Acad. Sci. USA.

[26] Stein BS, Bensch KG, Sussman HH (1984). Complete inhibition of transferrin recycling by monensin in K562 cells. J. Biol. Chem..

[27] Bhatt DH (2004). Cyclic AMP-Induced Repair of Zebrafish Spinal Circuits. Science (80-.)..

[28] Hiromura M (2004). Inhibition of Akt kinase activity by a peptide spanning the βA strand of the proto-oncogene TCL1. J. Biol. Chem..

[29] Kano F, Sako Y, Tagaya M, Yanagida T, Murata M (2000). Reconstitution of brefeldin A-induced golgi tubulation and fusion with the endoplasmic reticulum in semi-intact chinese hamster ovary cells. Mol. Biol. Cell.

