# Supplementary material for: LLO-mediated Cell Resealing System for Analyzing Intracellular Activity of Membrane-impermeable Biopharmaceuticals of Mid-sized Molecular Weight
Source: Sci Rep. 2018 Jan 31;8:1946. doi: 10.1038/s41598-018-20482-2 (PMC5792490; doi:10.1038/s41598-018-20482-2)

# SUPPLEMENTARY INFORMATIONS

## LLO-mediated Cell Resealing System for Analyzing Intracellular Activity of Membrane-impermeable Biopharmaceuticals of Mid-sized molecular Weight

Masataka Murakami[1], Fumi Kano[1,2], *Masayuki Murata[1,2,3]

[1]Department of Life Sciences, Graduate School of Arts and Sciences, The University of Tokyo, 3-8-1 Komaba, Meguro-ku, Tokyo, 153-8902, Japan
[2]Cell Biology Center, Institute of Innovative Research, Tokyo Institute of Technology, 4259 Nagatsuta, Midori-ku, Yokohama, Kanagawa, 226-8503, Japan
[3]Laboratoty of Frontier Image Analysis, Graduate School of Arts and Science, The University of Tokyo, 3-8-1 Komaba, Meguro-ku, Tokyo 153-8902, Japan

# Supplementary Figure 1

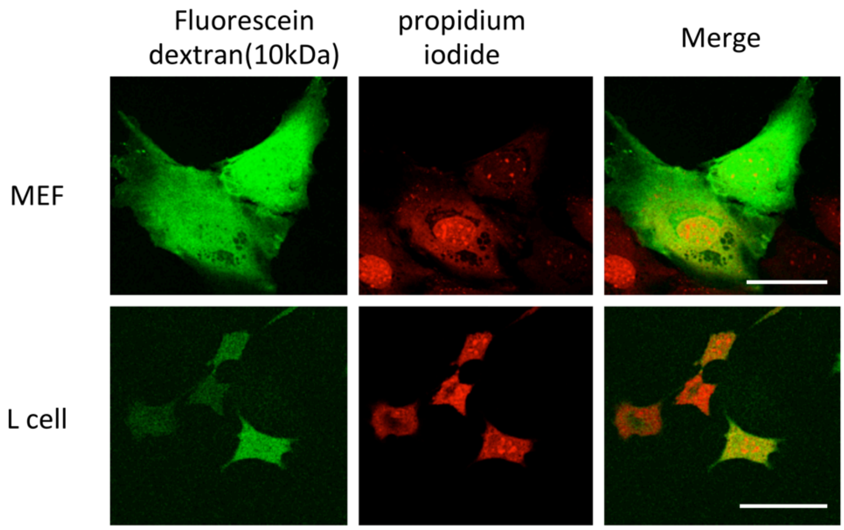


***Figure. S1 LLO-type resealing of MEF and L cells.***

Mouse embryonic fibroblasts (MEF) or mouse methylcholanthrene-induced sarcoma cells (L-cell) were incubated with 0.23 or 0.11 μg/ml LLO respectively, and were incubated for 10 min at 37°C in TB that contained propidium iodide. The cells were incubated with resealing buffer that contained 10 kDa dextran conjugated with fluorescein at 37°C for 30 min. After incubation with 1 mM $CaCl_2$ for 5 min, the cells were incubated with medium at 37˚C for 1 h and observed using confocal microscopy. Bar = 50 μm.

# Supplementary Figure 2

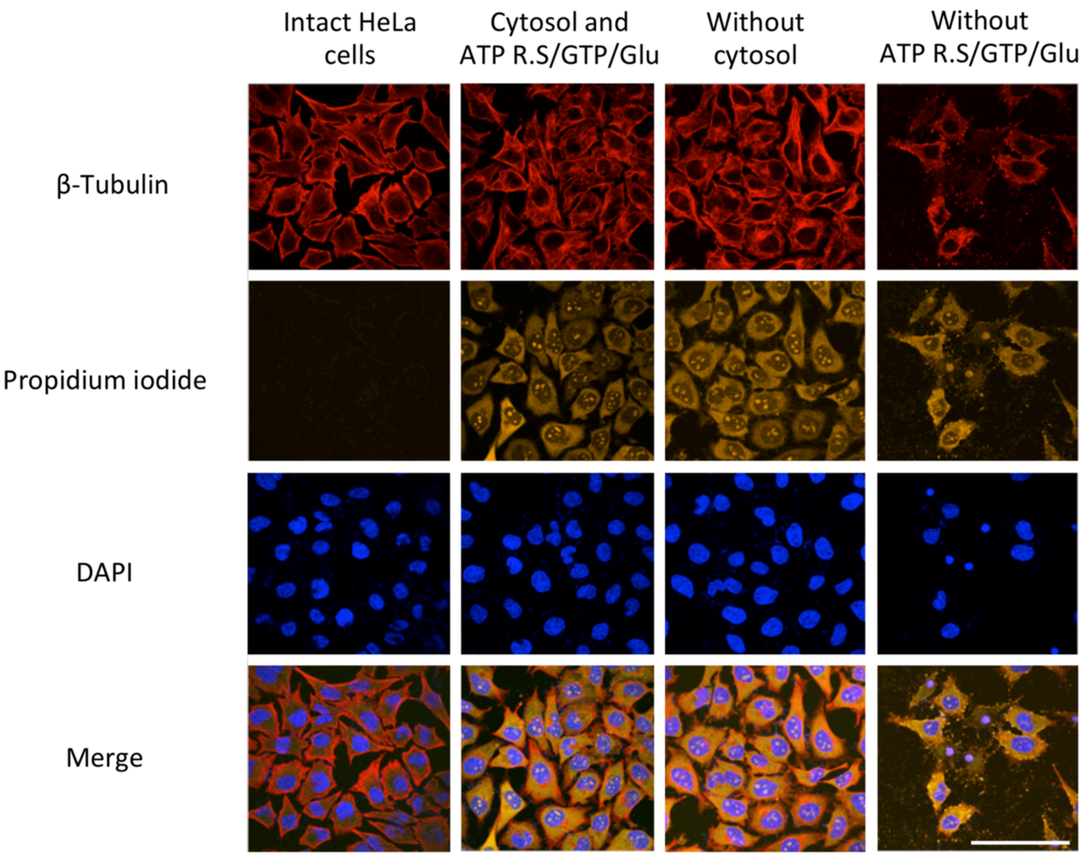


***Figure. S2 Dependency of LLO-type cell-resealing on ATP regeneration system, GTP and glucose.***

Semi-intact HeLa cells, that were stained with propidium idodide, were incubated with L5178Y cytosol, an ATP regeneration system, GTP and glucose (Cytosol and ATP R.S/GTP/Glu), ATP regeneration system, GTP, and glucose (Without cytosol), or L5178Y cytosol (Without ATP R.S/GTP/Glu) at 37˚C for 30 min. The cells were fixed with 4 % paraformaldehyde in PBS for 20 min, and then permeabilized with 0.2 % Triton X-100 in PBS for 15 min at room temperature. After blocking with 3 % BSA for 30 min, the cells were stained with mouse anti-β Tubulin antibody and DAPI (5 μg/ml). The cells were observed using confocal microscopy. Bar = 100 μm.

# Supplementary Figure 3

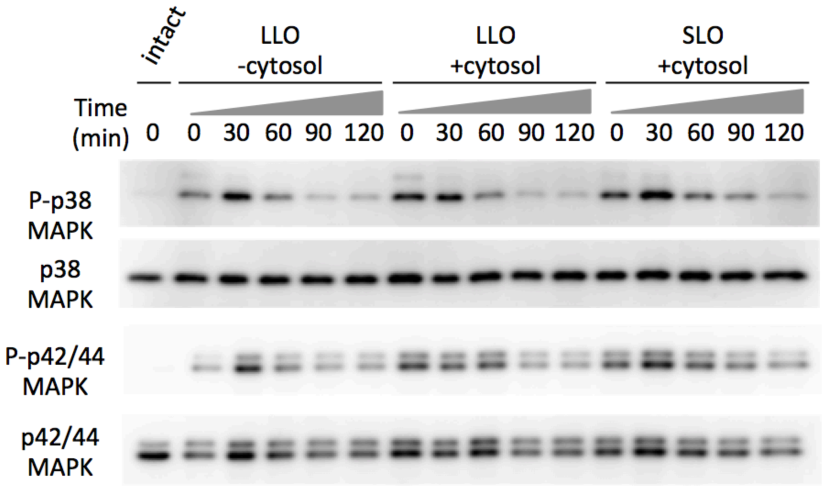


***Figure. S3 Phosphorylation status of p38 MAPK and p42/44 MAPK in LLO-type or SLO-type resealed cells.***

LLO- or SLO-type resealed HeLa cells that contained resealing buffer with (+cytosol) or without (-cytosol) cytosol were prepared as described in Fig. 2B. The cells were incubated with medium at 37˚C for 0, 30, 60, 90, 120 min, lysed, and were subjected to Western blotting using antibodies against p38 MAPK, phosphorylated p38 MAPK, p42/44 MAPK, and phosphorylated p42/44 MAPK.

# Supplementary Figure 4

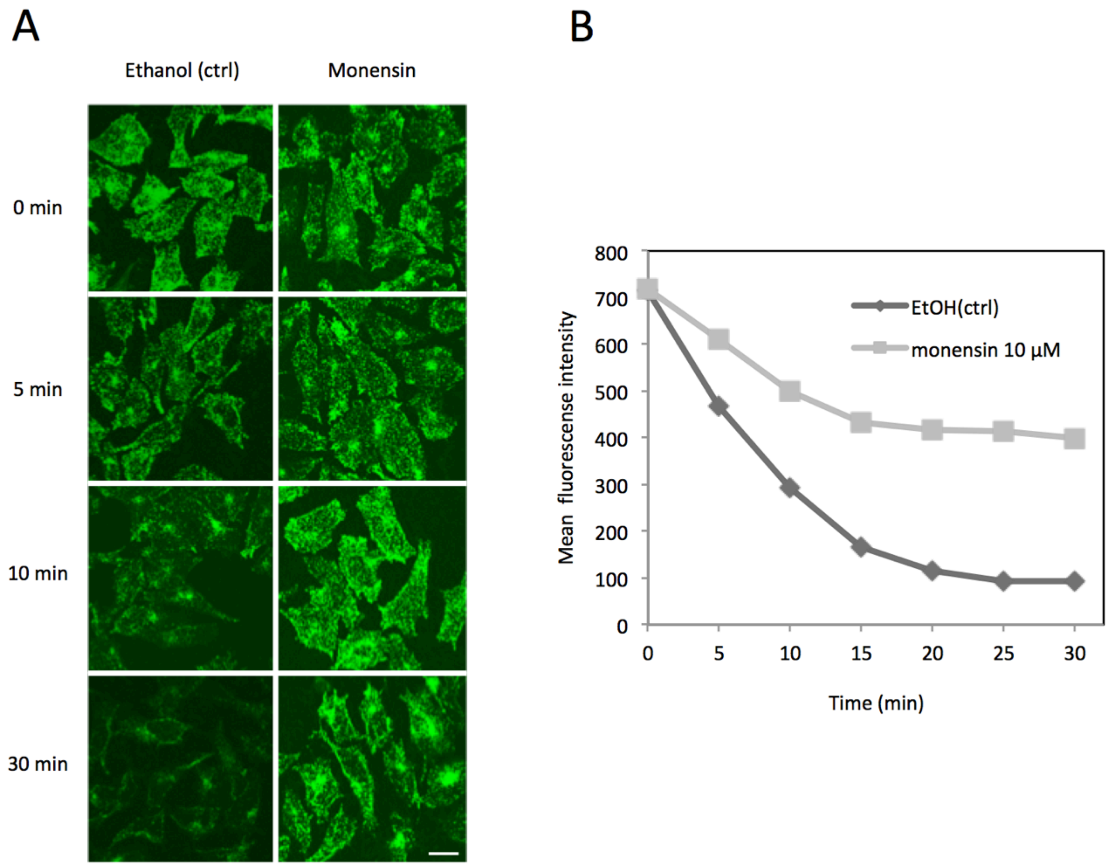


***Fig. S4 Recycling of transferrin in LLO resealed cells.***

HeLa cells were permeabilized with 0.15 μg/ml LLO. After resealing with 1 mM $CaCl_2$, the cells were incubated with medium for 4 h. After washing with PBS twice, the cells were incubated with 10 μg/ml alexa 488 conjugated human transferrin (molecular probes) in serum free medium for 30 min at 37℃ to allow uptake and delivery to endosomal compartments, and then chased in medium contained excess non-labeled transferrin (Roche) in the presence or absence of 10 μM monensin (SIGMA) for various periods of time. The cells were washed twice with PBS, and were observed by using a confocal microscope or were subjected to flow cytometry. (A) Typical images of the cell at each time. Bar = 20 μm. (B) Mean fluorescence intensity of the cell at each time point was indicated in graph.

# Supplementary Figure 5

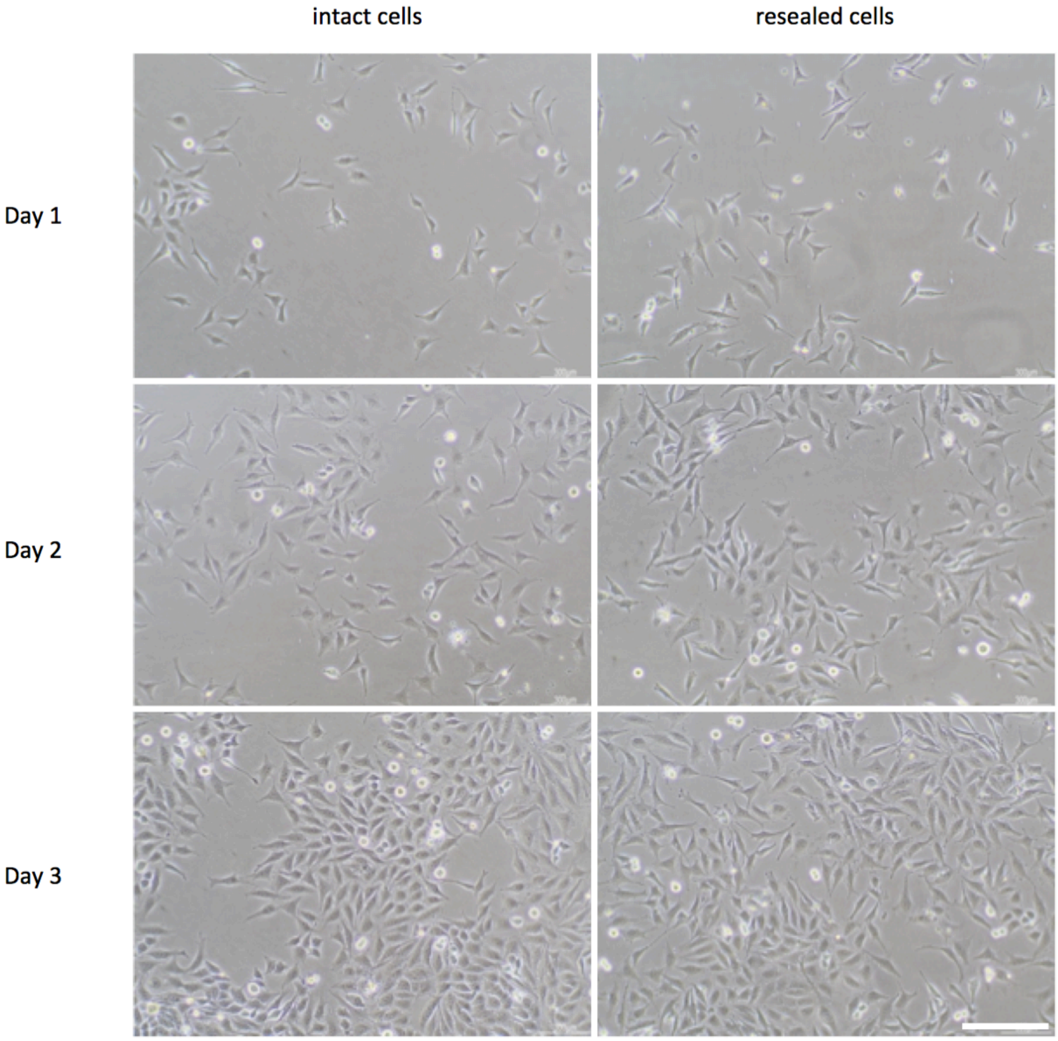


***Figure. S5 Proliferation of LLO-type resealed cells.***

HeLa cells were permeabilized with 0.15 μg/ml LLO and incubated with resealing buffer containing ATP regeneration system, GTP and glucose for 30 min. After resealing with 1 mM $CaCl_2$, the cells were incubated with medium at 37℃ 5% $CO_2$ for overnight. Then, intact or resealed HeLa cells were seeded at $3\times10^4$ cell/well at 24 well plate, and incubated with medium for 1, 2 or 3 days. The cells were observed by bright field microscopy. Bar = 200 μm.

# Supplementary Figure 6

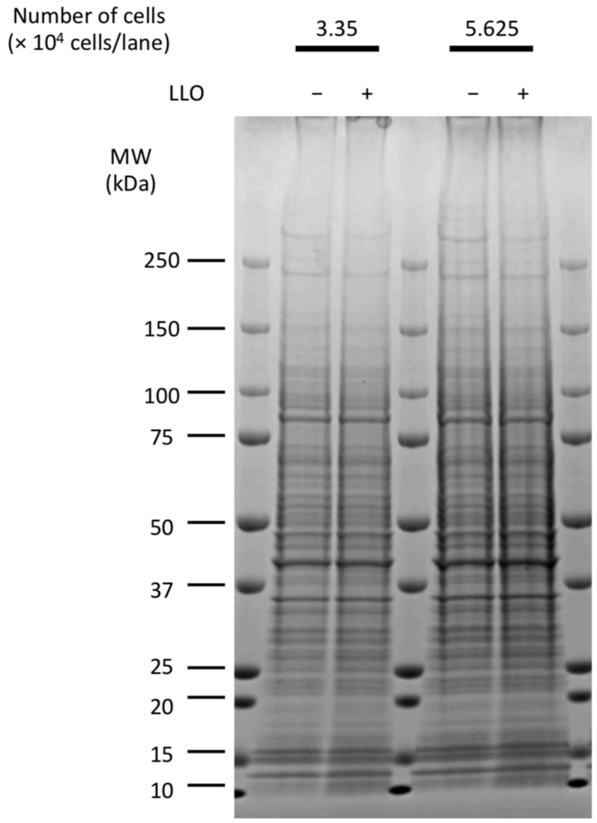


***Fig. S6 Coomassie blue staining of total protein prepared from intact and LLO-type resealed cells.***

HeLa cells were incubated with or without 0.15 µg/ml LLO on ice for 5 min. After washing with TB three times, the cells were incubated in preheated TB at 37℃ for 10 min. After lysing the cells, total protein was separated using SDS-PAGE and subjected to CBB staining. The number of cells per well is indicated at the top of the figure. No significant difference in the protein band pattern was observed, indicating that the composition of the proteins appeared similar.

# Supplementary Figure 7

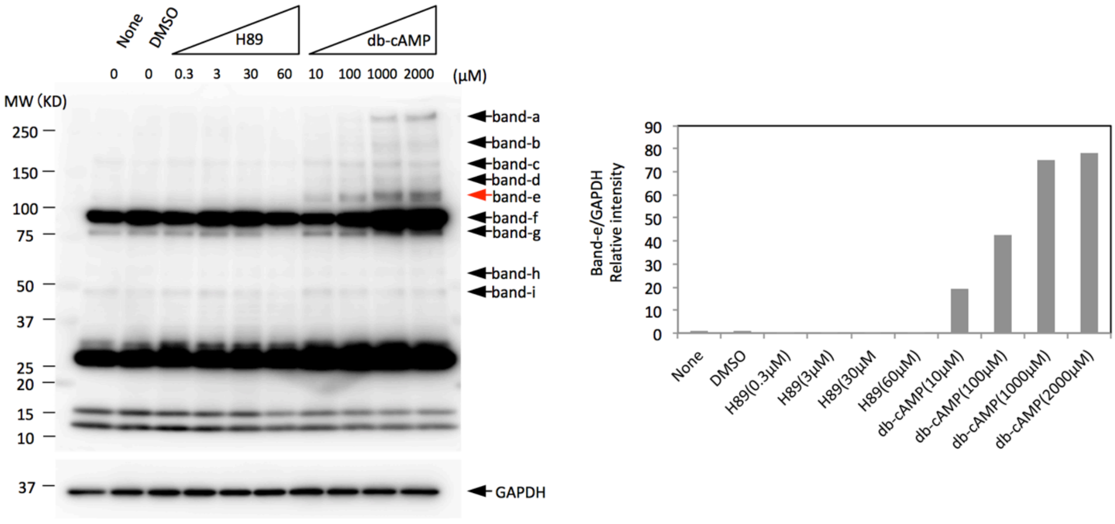


***Figure. S7 Band e is a sensitive PKA substrate read-out of cAMP stimulation***. HeLa cells were incubated with DMSO, 0.3, 3, 30 or 60 μM H89 or 10, 100, 1000, 2000 μM db-cAMP in medium at 37℃ for 60 min. The cells were lysed, and were subjected to Western blotting using antibodies against Phospho-PKA substrate and GAPDH. The arrows (band-a to -i) indicate the bands, the intensity of which were increased in dependence on the db-cAMP concentration. Band e is the band that increased its intensity the most following db-cAMP treatment. The relative intensity of band e to GAPDH is shown in the right graph.

# Supplementary Figure 8

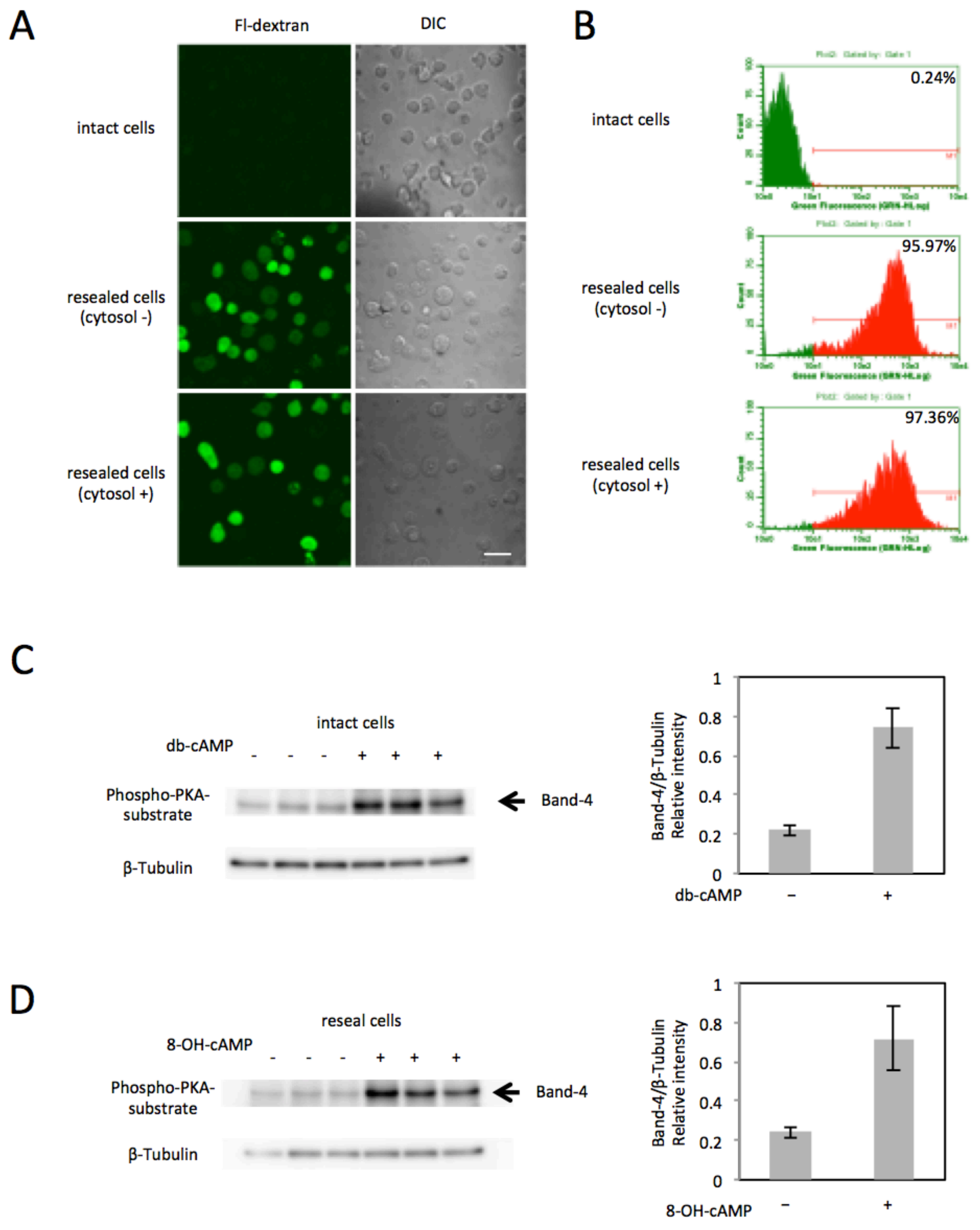


*Figure. S8 LLO-type cells resealing of non-adherent murine lymphoma-derived EL4 cells and in vivo fuctional analysis of membrane impermeable cAMP analog in LLO-type resealed EL4 cells.*

(A, B) $2\times10^6$ of EL4 cells were suspended in RPMI medium without serum and centrifuged at 300 g for 5 min at 4˚C. The pelleted cells were resuspended in 0.15 μg/ml LLO in RPMI and rotated at 4˚C for 12 min. After washing the cells by centrifugation and the subsequent suspension of the cell pellet in RPMI medium without serum three times, the cells were resuspended and incubated with pre-warmed TB at 37˚C for 10 min to permeabilize the cell membrane. The cells were centrifuged and the cell pellet was suspended in TB, and the washing process was repeated once. The pellet of permeabilized EL4 cells were suspended and incubated with resealing buffer that contained 3 kDa fluorescein-dextran in the presence or absence of 1.5 mg/ml L5178Y cytosol at 37˚C for 30 min. After the addition of $CaCl_2$ to the final concentration of 1 mM and the further incubation at 37˚C for 5 min to repair the lesion of cell membrane, RPMI medium was added and centrifuged at 300 g for 5 min. The resealed EL4 cells were washed with medium by centrifugation and the subsequent resuspension with medium once, and were incuabated at 37˚C 5 % $CO_2$ for 30 min. Then the cells were subjected to flow cytometry, which measures the fluorescent intensity of fluorescein-dextran in each resealed EL4 cells (B), and the fluorescent microscopic observation (A). Percentage of fluorescein positive cell is indicated in histogram (B). (C) EL4 cells were incubated with or without 1 mM db-cAMP in medium at 37℃ for 60 min. The cells were lysed, and were subjected to Western blotting using antibodies against Phospho-PKA substrate and β-Tubulin. The arrows indicate the band that the intensity of which were increased in dependence on the db-cAMP concentration. Band 4 is the band that increased its intensity the most following db-cAMP treatment. The relative intensity of band e to β-Tubulin is shown in the right graph. (D) After permeabilizing EL4 cells as described (A), the semi-intact EL4 cells were incubated with resealing buffer with or without 1 mM 8-OH-cAMP, which is a membrane-permeable cAMP analogue, at 37˚C for 30 min. the cells were resealed by addition of 1 mM $CaCl_2$ at 37˚C for 5 min, and further incubated with medium at 37˚C at 5 % for 1 h. The cells were lysed and were subjected to Western blotting using anti-phospho-PKA-substrate antibody and anti-β-tubulin antibody. The relative intensity of band 4 to β-Tubulin is shown in the right graph.

# Full-length gels from Figures

## Full-length gels from Figure 6A:

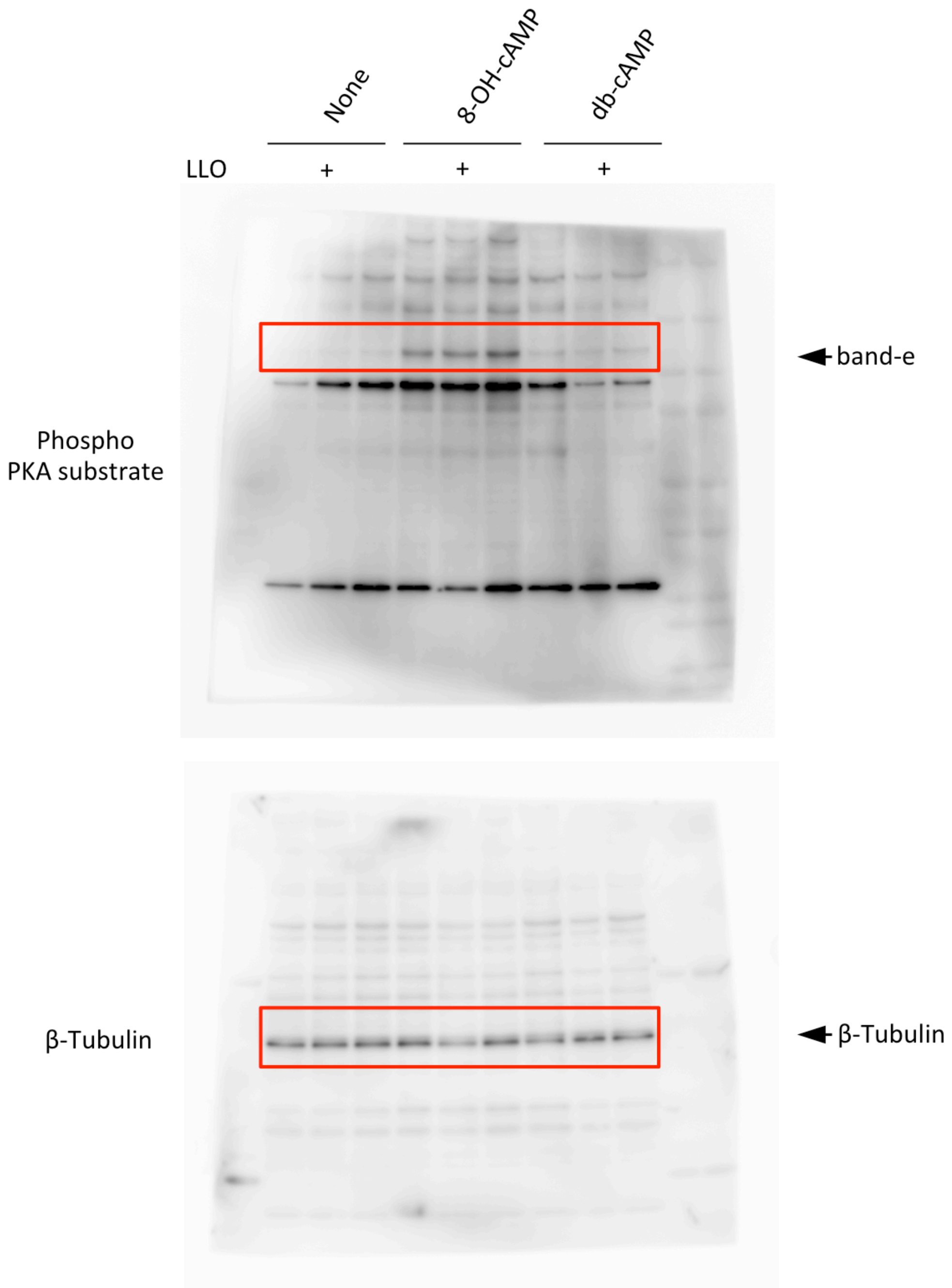

# Full-length gels from Figure 6C:

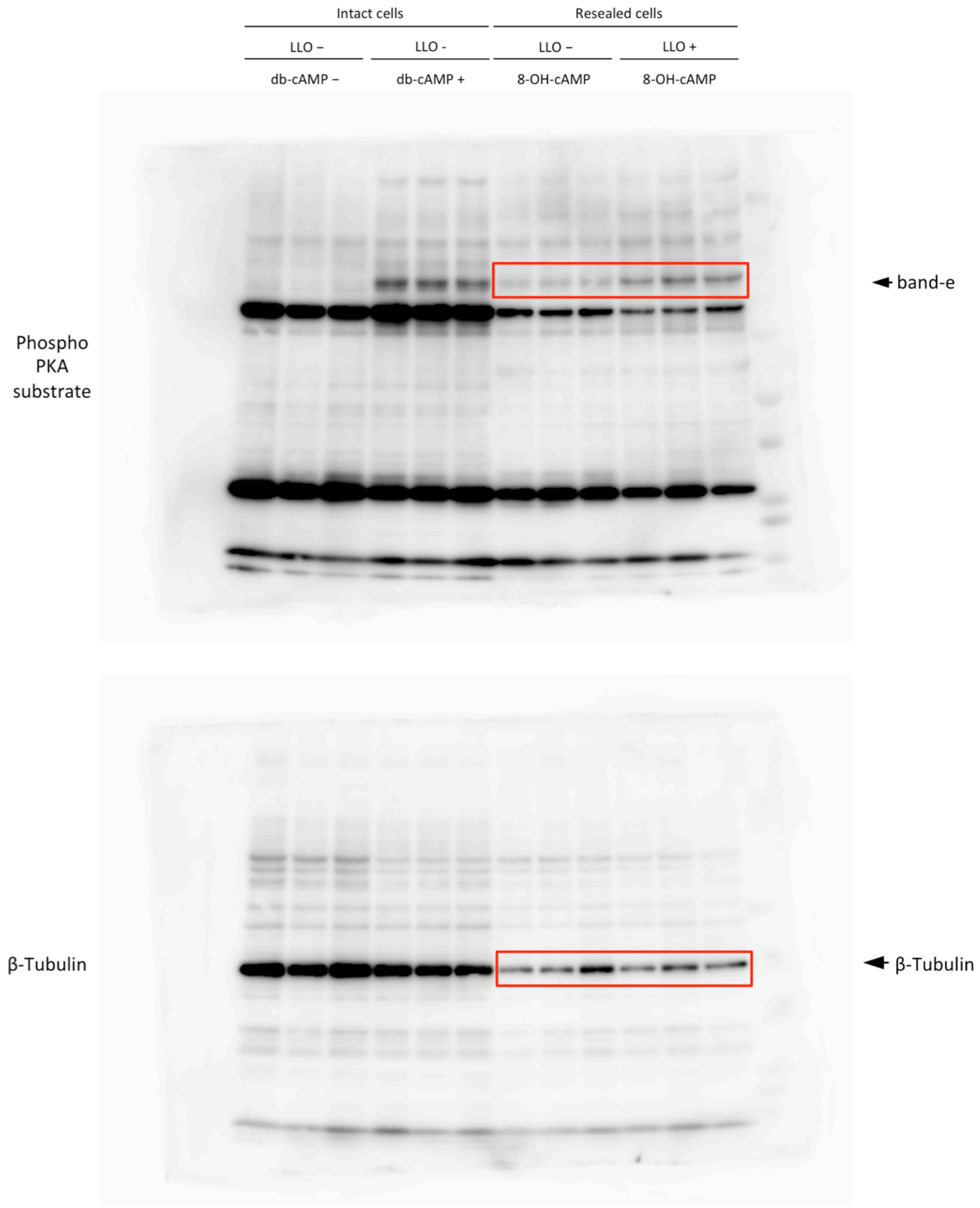

# Full-length gels from Figure 7B:

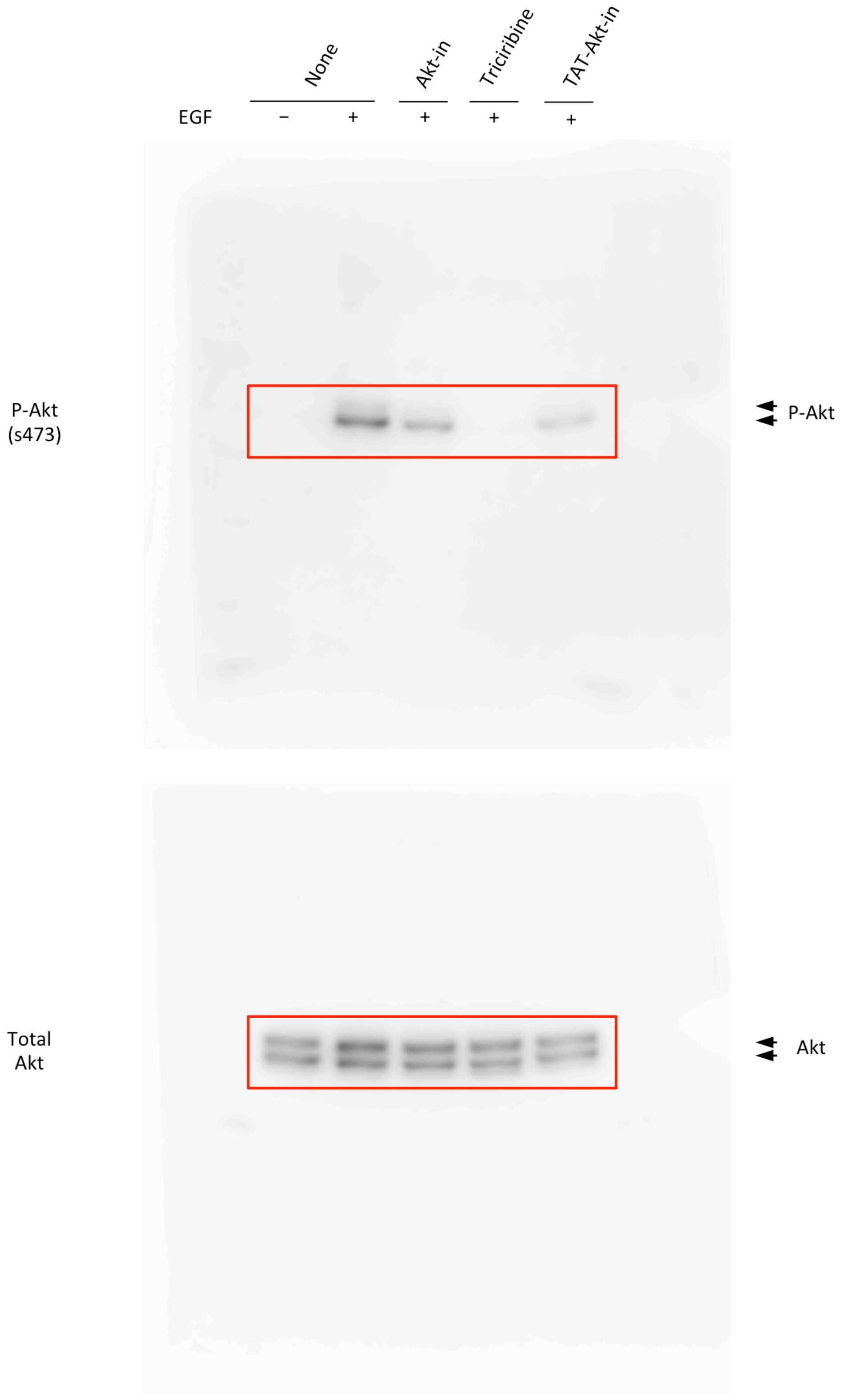

# Full-length gels from Figure S8:

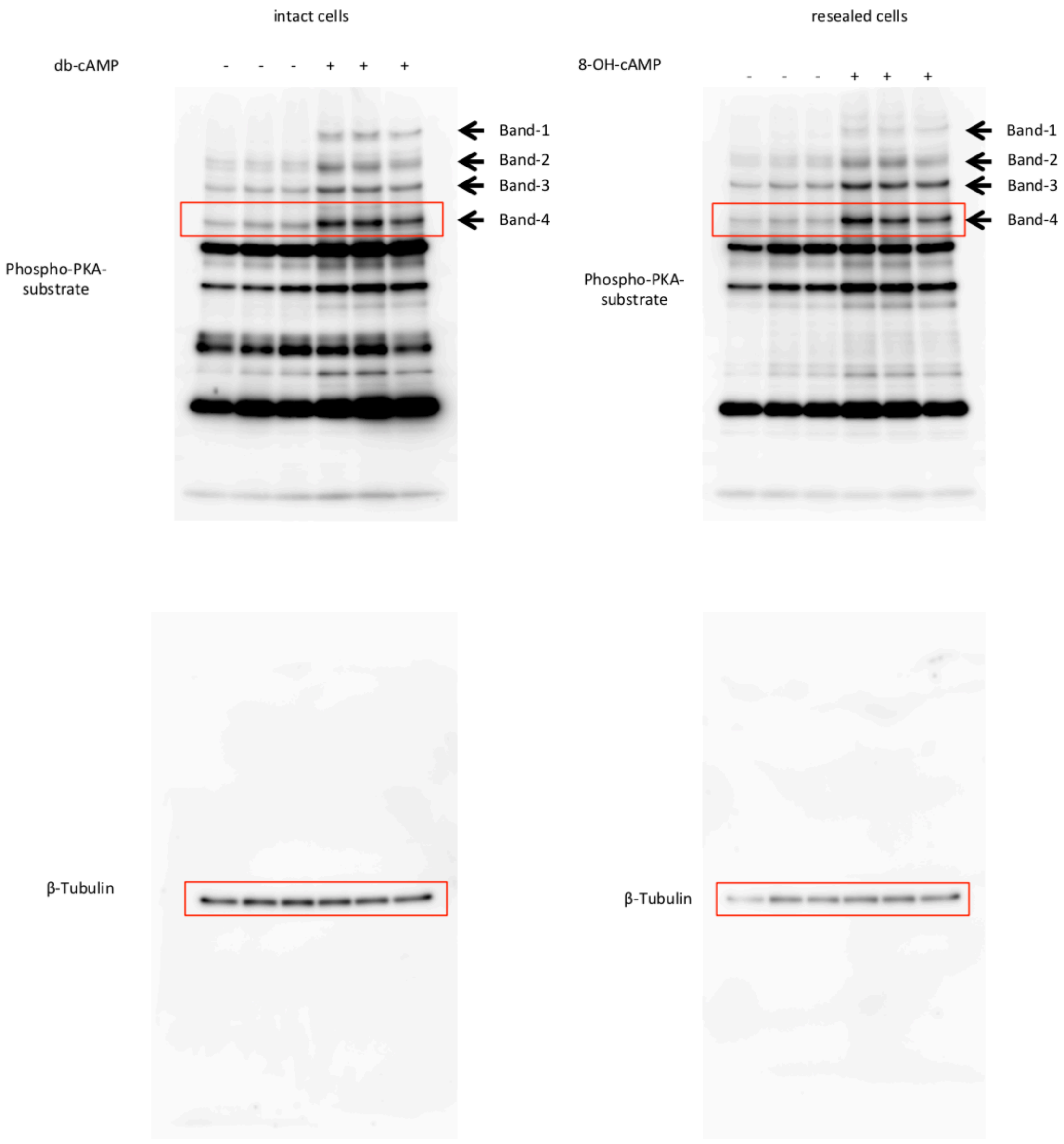

Supplement: Supplementary file 1 — Supplementary Information [file 41598_2018_20482_MOESM1_ESM.pdf]
